# Genome‐wide meta‐analysis of SNP and antihypertensive medication interactions on left ventricular traits in African Americans

**DOI:** 10.1002/mgg3.788

**Published:** 2019-08-13

**Authors:** Anh N. Do, Wei Zhao, Abigail S. Baldridge, Laura M. Raffield, Kerri L. Wiggins, Sanjiv J. Shah, Stella Aslibekyan, Hemant K. Tiwari, Nita Limdi, Degui Zhi, Colleen M. Sitlani, Kent D. Taylor, Bruce M. Psaty, Nona Sotoodehnia, Jennifer A. Brody, Laura J. Rasmussen‐Torvik, Donald Lloyd‐Jones, Leslie A. Lange, James G. Wilson, Jennifer A. Smith, Sharon L. R. Kardia, Thomas H. Mosley, Ramachandran S. Vasan, Donna K. Arnett, Marguerite R. Irvin

**Affiliations:** ^1^ Department of Epidemiology University of Alabama at Birmingham Birmingham Alabama USA; ^2^ Department of Epidemiology University of Michigan Ann Arbor Michigan USA; ^3^ Feinberg School of Medicine Northwestern University Chicago Illinois USA; ^4^ Department of Genetics University of North Carolina Chapel Hill North Carolina USA; ^5^ Cardiovascular Health Research Unit, Department of Medicine University of Washington Seattle Washington USA; ^6^ Department of Biostatistics University of Alabama at Birmingham Birmingham Alabama USA; ^7^ Department of Neurology University of Alabama at Birmingham Birmingham Alabama USA; ^8^ School of Biomedical Informatics University of Texas Health Sciences Center at Houston Houston Texas USA; ^9^ Cardiovascular Health Research Unit, Department of Medicine University of Washington Seattle Washington USA; ^10^ Department of Pediatrics, The Institute for Translational Genomics and Population Sciences LABioMed at Harbor‐UCLA Medical Center Seattle Washington USA; ^11^ Cardiovascular Health Research Unit, Departments of Medicine, Epidemiology and Health Services University of Washington Seattle Washington USA; ^12^ Kaiser Permanente Washington Health Research Institute Seattle Washington USA; ^13^ Cardiovascular Health Research Unit, Division of Cardiology, Departments of Medicine and Epidemiology University of Washington Seattle Washington USA; ^14^ Department of Preventive Medicine Feinberg School of Medicine Northwestern University Chicago Illinois USA; ^15^ Department of Medicine University of Colorado Denver Aurora Colorado USA; ^16^ Department of Physiology and Biophysics University of Mississippi Medical Center Jackson Mississippi USA; ^17^ Department of Medicine University of Mississippi Medical Center Jackson Mississippi USA; ^18^ Departments of Medicine and Preventive Medicine Boston University School of Medicine Boston Massachusetts USA; ^19^ College of Public Health University of Kentucky Lexington Kentucky USA

**Keywords:** antihypertensive treatment, GWAS, left ventricular trait, pharmacogenetics

## Abstract

**Background:**

Left ventricular (LV) hypertrophy affects up to 43% of African Americans (AAs). Antihypertensive treatment reduces LV mass (LVM). However, interindividual variation in LV traits in response to antihypertensive treatments exists. We hypothesized that genetic variants may modify the association of antihypertensive treatment class with LV traits measured by echocardiography.

**Methods:**

We evaluated the main effects of the three most common antihypertensive treatments for AAs as well as the single nucleotide polymorphism (SNP)‐by‐drug interaction on LVM and relative wall thickness (RWT) in 2,068 participants across five community‐based cohorts. Treatments included thiazide diuretics (TDs), angiotensin converting enzyme inhibitors (ACE‐Is), and dihydropyridine calcium channel blockers (dCCBs) and were compared in a pairwise manner. We performed fixed effects inverse variance weighted meta‐analyses of main effects of drugs and 2.5 million SNP‐by‐drug interaction estimates.

**Results:**

We observed that dCCBs versus TDs were associated with higher LVM after adjusting for covariates (*p* = 0.001). We report three SNPs at a single locus on chromosome 20 that modified the association between RWT and treatment when comparing dCCBs to ACE‐Is with consistent effects across cohorts (smallest *p* = 4.7 × 10^−8^, minor allele frequency range 0.09–0.12). This locus has been linked to LV hypertrophy in a previous study. A marginally significant locus in *BICD1* (rs326641) was validated in an external population.

**Conclusions:**

Our study identified one locus having genome‐wide significant SNP‐by‐drug interaction effect on RWT among dCCB users in comparison to ACE‐I users. Upon additional validation in future studies, our findings can enhance the precision of medical approaches in hypertension treatment.

## 1 INTRODUCTION

Left ventricular (LV) hypertrophy (LVH) is the thickening of the myocardium of the left ventricle of the heart. LVH is common in the general population (16% of European Americans [EAs], and up to 43% of African Americans [AAs]) and even more prevalent among individuals with hypertension (up to 60%). LVH is recognized as an independent risk factor for cardiovascular outcomes including stroke, heart failure and all‐cause mortality (Benjamin & Levy, 1999; Schillaci et al., 2000; Vakili, Okin, & Devereux, 2001). Indeed, LVH may be a better predictor of mortality than coronary artery disease in many populations (Liao, Cooper, McGee, Mensah, & Ghali, 1995). Additionally, LVH poses greater cardiovascular risk to AAs than to other ethnic groups (Havranek et al., 2008; Kizer et al., 2004).

Antihypertensive treatments have been reported to decrease LV mass (LVM) independently of their blood pressure lowering effects in participants treated for hypertension (Gosse et al., 2000; Klingbeil, Schneider, Martus, Messerli, & Schmieder, 2003; Mathew et al., 2001). However, there is a lack of consensus on the most effective antihypertensive agents for decreasing LVM (Aurigemma et al., 2003; Fagard, Celis, Thijs, & Wouters, 2009; Klingbeil et al., 2003). There are likely subgroups of patients who may benefit more from using a specific class of antihypertensive drugs. Additionally, the effect of antihypertensive agents on LV diastolic function is also controversial (Aurigemma et al., 2003; Fagard et al., 2009; Schmieder, Martus, & Klingbeil, 1996). Furthermore, despite their increased susceptibility to LVH sequelae, AAs have been underrepresented in previous studies on the effect of antihypertensive drugs on LVM (Aurigemma et al., 2003; Fagard et al., 2009; Klingbeil et al., 2003).

Though studies suggest that antihypertensive agents may regress LVM, follow‐up studies have shown that there is considerable interindividual variation in antihypertensive treatment responses, suggesting that genetic factors may contribute to such differences (He et al., 2005; Kohno et al., 1999; Liljedahl et al., 2004). Previous candidate gene studies have attempted to find pharmacogenetic factors associated with LVH and related traits (He et al., 2005; Liljedahl et al., 2004). However, most previous studies had small sample sizes, considered few genetic variants and their results were not replicated (He et al., 2005; Kohno et al., 1999; Liljedahl et al., 2004). Genome‐wide scans for variants that may modify the effect of antihypertensive treatment class on LV traits in a sizable population of AAs may help identify novel pharmacogenetic variants.

To fill the research gaps noted above, this study combines both pharmacoepidemiologic (main effects) and pharmacogenetic analyses. We first evaluated the relationship between the three most common antihypertensive treatments for AAs (thiazide and related diuretics [TDs], angiotensin converting enzyme inhibitors [ACE‐Is], and dihydropyridine calcium channel blockers [dCCBs]) and quantitative traits related to LVH measured by echocardiography using a cross‐sectional design. Next, we examined how the antihypertensive drug‐trait relationship may be modified by genomic variants using data collected from AA participants of five observational epidemiology studies from the Cohorts for Heart and Aging Research in Genomic Epidemiology consortium (Psaty et al., 2009).

## 2 METHODS AND MATERIALS

### 2.1 Study population

Five observational epidemiology studies with AAs including the Coronary Artery Risk Development in Young Adult Study (CARDIA), the Cardiovascular Health Study (CHS), the Jackson Heart Study (JHS), the Genetic Epidemiology Network of Atherosclerosis Study (GENOA), and the Hypertension Genetic Epidemiology Network (HyperGEN) contributed data on antihypertensive treatment, echocardiographic traits, and genome‐wide association study (GWAS) data for the current investigation. Guidelines on collaboration, phenotype harmonization, covariate selection, and the analysis plan for both within‐cohort GWA and meta‐analysis of results across studies were adopted by each cohort. Additionally, each cohort obtained approval from the respective institutional review boards for informed consent procedures, examination and surveillance components, and data security measures. We included 2,068 AAs treated for hypertension in the current meta‐analysis. Further details of the study population are provided in Supporting Information.

### 2.2 Echocardiographic phenotypes

The study assessed two LV structures as primary outcomes, that is, LVM and relative wall thickness (RWT). In all five cohort studies, LVM was calculated using the American Society of Echocardiography corrected formula by Devereux et al.: 0.80 × 1.04 × [(IVSDD + PWTD + LVIDD)^3^ − LVIDD^3^] + 0.6 g in which IVSDD is the interventricular septum thickness, LVIDD is the LV internal dimension at end‐diastole, and PWTD is the thickness at end‐diastole of the LV posterior wall (Lang et al., 2015). RWT was calculated as twice the PWTD divided by the LVIDD (Devereux et al., 1986). Both LVM and RWT were available in all five cohort studies.

Secondary outcomes included two LV diastolic function measures and a speckle tracking trait. LV diastolic function measures included early diastolic tissue velocity at the septal mitral annulus (*e*′ velocity) and the ratio of early (*E*) transmitral flow velocity to early diastolic tissue velocity at the septal mitral annulus (*E*/*e*′ ratio). *e*′ velocity and *E*/*e*′ ratio correlate with LV relaxation and LV diastolic filling pressure, respectively. Better cardiac function is indicated by low *E*/*e*′ ratio and high *e*′ velocity. LV global longitudinal strain (GLS) is a measure of LV systolic function assessed by speckle tracking. GLS describes the relative length change of the LV myocardium between end‐diastole and end‐systole (Lang et al., 2015). Higher absolute GLS indicates better cardiac function (Lang et al., 2015). GLS has been strongly associated with cardiovascular mortality (Kramann et al., 2014; Selvaraj et al., 2014). Each of *e*′ velocity, *E*/*e*′ ratio, and GLS data were available in the CARDIA and HyperGEN studies only.

### 2.3 Definition of drug exposure

We compared three antihypertensive classes of drugs (ACE‐Is, dCCBs, and TDs) in a pairwise manner in the following three statistical models: Model 1) ACE‐I use versus TD use (reference = TD use) where ACE‐I exposure was defined as the use of an ACE‐I in a single or combination preparation without concomitant use of a TD versus TD exposure without ACE‐I; Model 2) dCCB use versus TD use (reference = TD use) where dCCB exposure was defined as the use of a dCCB in a single or combination preparation without concomitant use of a TD versus TD exposure without dCCB; Model 3) dCCB use versus ACE‐I use (reference = ACE‐I use) where dCCB exposure was defined as the use of a dCCB in a single or combination preparation without concomitant use of an ACE‐I versus ACE‐I exposure without dCCB. Drug groupings were based on manually curated lists reviewed by experts from each cohort study to include all relevant antihypertensive drugs from the United States. Antihypertensive drug exposure was assessed by medication inventory or self‐report in each of the five cohort studies (see Supporting Information). In our approach, participants taking more than one medication class may contribute data to more than one model. A medication inventory of eligible antihypertensive treatments for each model and excluded treatments is provided in Supporting Information List 1 and List 2, respectively.

### 2.4 Genotyping and imputation

Genome‐wide single nucleotide polymorphism (SNP) genotyping was performed within each study using Illumina or Affymetrix genotyping arrays. SNP quality control (QC) was performed prior to imputation using PLINK, Birdseed v1.33, or Illumina GenomeStudio. QC measures removed (a) samples with genotyping success rate < 95%, (b) SNPs failing genotyping call rate thresholds, between 90% and 99%, (c) monomorphic SNPs, (d) SNPs that mapped to several loci in the human genome, and (e) SNPs with minor allele frequency (MAF) <1%. Other QC filters included removing SNPs (a) with Mendelian inconsistencies (for cohorts with family data), and (b) those with significant deviation from Hardy–Weinberg equilibrium with *p*‐value < 10^−6^ in JHS and HyperGEN or <10^−5^ in CHS. A combined YRI and CEU reference panel from HapMap phase 2 (build 36 release 22) was used for imputation in each of the five cohorts, as the African‐American population is admixed with ~17%–19% European ancestry (Zhu et al., 2005). More details of genotyping, QC, and imputation for each study are provided in Table S1.

### 2.5 Statistical analysis for main effect of antihypertensive medication on LV traits

#### 2.5.1 Statistical analysis of drug effects on LV traits within studies

Each study independently implemented a predefined analysis plan. All cohorts excluded extreme values (>5 *SD*s of its mean) for each echocardiographic measure. Natural log‐transformations were made for LVM, RWT, and *E*/*e*′ ratio to satisfy model distributional assumptions. For cohorts of unrelated individuals, we used linear regression models (CARDIA, CHS) or generalized estimating equations with a sandwich estimator of the variance (JHS). For family‐based cohorts (HyperGEN and GENOA), we used mixed effects models. The models tested the main effect of antihypertensive treatment class (ACE‐I vs. TD; dCCB vs. TD; dCCB vs. ACE‐I, as described in Definition of drug exposure) on each of the LV traits separately. Each model was adjusted for age, sex, weight, height, count of antihypertensive treatment classes, estimated glomerular filtration rate, and Type 2 diabetes. Study site and/or other study specific variables were included as covariates as needed (e.g., center in HyperGEN). Family relatedness information was used as a random effect in HyperGEN and GENOA. The models for *e*′ velocity, *E*/*e*′ ratio, and GLS were additionally adjusted for institution, reader, and image quality to control for interobserver variability.

#### 2.5.2 Meta‐analysis of drug effects on LV traits

We performed fixed effects inverse variance weighted meta‐analysis using the METASOFT software, where the weights were calculated as the reciprocal of estimated variance (*SE*) of the effect size (β) from each study. If heterogeneity was observed (*p*‐value of Cochran's *Q* statistic < 0.05), we reported results of a random effect model where between‐study variance of heterogeneity was used as a weight for the random effect. A *p*‐value < 0.016 was considered significant (α = 0.05/3, correcting for three pairwise comparisons).

#### 2.5.3 Pharmacogenetic analyses within studies

Pharmacogenetic GWAS models were identical to the main effect models described above, except for the addition of SNPs (under an additive model) and SNP‐by‐drug interaction terms as well as additional adjustment for ancestry using principal components. A sensitivity model adjusting for the other medication class (e.g., adjusting for TD in the model of dCCB vs. ACE‐I) as opposed to the count of antihypertensive treatment classes was conducted in the HyperGEN AA study.

#### 2.5.4 Pharmacogenetic meta‐analysis

Prior to the meta‐analysis, we verified strand alignment across studies by comparing each SNP in each study to the same SNP in 1,000 Genome phase 3. The GWAS data were aligned to the forward strand in each study. To control inflation for poorly calibrated tests for less frequent variants among less common drug exposures we calculated the SNP‐specific filter degrees of freedom (*df*) for each cohort as the product of the number of drug‐exposed participants (i.e., the number of nonreference drug exposure), the SNP imputation quality (range: 0, 1), and the MAF (range: 0, 0.50) (Marwick et al., 2015). We excluded cohort‐specific results for SNPs with *df* < 10. Genomic control was applied to all studies (Devlin & Roeder, 1999). We restricted our meta‐analysis to autosomal SNPs available in at least two studies. We used study‐specific interaction estimates (β) and “corrected” *SE* in fixed effects inverse variance weighted meta‐analysis using METAL software. To obtain “corrected” *SE*, *p*‐values were recalculated by applying a reference *t*‐distribution for the ratio of the SNP‐by‐drug estimates (β) to its *SE*. Corrected *SE*s were used for the *t*‐distribution‐based *p*‐values when assuming a normal distribution for the ratio of the SNP‐by‐drug estimates (β) to its corrected *SE*. Such correction was necessary due to known underestimation of *SE*s by robust methods when any SNP‐treatment stratum is small. The cohort specific *df* for the *t* reference distribution was estimated using Satterthwaite's method in cohorts with unrelated participants. In HyperGEN and GENOA, *df* was estimated as the filter *df* described above. The genome‐wide threshold for significant SNP‐by‐drug interaction was *p* < 5 × 10^−8^. SNPs with heterogeneity *p*‐value > 0.05 were excluded.

### 2.6 Validating results in an external cohort

EAs in the HyperGEN study served as an external validation cohort. Similar to AAs from HyperGEN, LV structures were measured using the same protocol and images were read at the same echocardiography reading center (Williams et al., 2000). Identical inclusion and exclusion criteria to define drug exposure were applied to identify 613 EAs treated for hypertension with relevant data for validation.

HyperGEN EAs were genotyped with the Affymetrix Genome‐Wide Human SNP Array 5.0 Array (Arnett et al., 2011). SNP QC was performed prior to imputation using PLINK. Low quality samples and SNPs were excluded using the same criteria applied for HyperGEN AAs. The CEU reference panel from HapMap phase 2 (build 36 release 22) was used for imputation. The same linear mixed effect models included the same covariates and family information as a matrix to adjust for familial relatedness. Top SNPs identified in the discovery cohort (Table 4) and SNPs in high linkage disequilibrium (*R* ^2^ ≥ 0.7 within 100 kb found using LDproxy; https://ldlink.nci.nih.gov/?tab=ldproxy) were used for validation.

## 3 RESULTS

### 3.1 Characteristics of study population

The characteristics of 2,068 participants from five cohorts are shown in Table 1. The participants were predominantly women, on average middle‐aged (mean age range = 50–74 years), and nondiabetic. LVM slightly varied across the five studies (mean range = 158.8–185.5 g), whereas RWT was similar.

**Table 1:** Characteristics of study participants (*N* = 2,068)

	CARDIA	CHS	JHS	GENOA	HyperGEN
Sample size	251	290	571	280	676
Age, year (*SD*) (year)	50.3 (3.6)	74.3 (5.2)	54.2 (10.6)	62.7 (9.56)	51.5 (10.4)
Female, *N* (%)	173 (68.9)	203 (70.0)	379 (66.4)	195 (69.6)	490 (72.5)
Height, m, mean (*SD*)	1.7 (0.1)	1.6 (0.09)	1.7 (0.095)	1.68 (0.09)	1.7 (0.08)
Weight, kg, mean (*SD*)	96.9 (25.6)	78.0 (14.0)	95.6 (22.3)	90.8 (19.5)	91.9 (22.7)
eGFR, ml min^−1^ 1.73 m^−2^, mean (*SD*)	101.0 (25.2)	80.1 (20.5)	92.4 (22.3)	87.8 (21.03)	89.9 (21.2)
Type 2 diabetes, *N* (%)	73 (29.3)	81 (27.9)	183 (32.1)	93 (33.2)	173 (25.6)
Echocardiographic measure					
LVM, g, mean (*SD*)	185.5 (61.5)	161.8 (60.1)	158.8 (43.1)	163.4 (44.8)	177.4 (49.4)
RWT, cm, mean (*SD*)	0.38 (0.09)	0.42 (0.08)	0.39 (0.06)	0.33 (0.05)	0.43 (0.05)
Drug exposure					
TDs, *N* (%)	123 (49.0)	45 (15.5)	239 (41.9)	161 (57.5)	172 (25.4)
Monotherapy, *N* (%)	16 (13.1)	17 (37.8)	30 (12.6)	24 (14.9)	68 (39.5)
Average number of ATH, mean (*SD*)	2.3 (0.9)	1.7 (0.7)	2.3 (0.9)	2.2 (0.8)	1.8 (0.7)
ACE‐Is, *N* (%)	109 (43.4)	53 (18.3)	223 (39.1)	138 (49.2)	208 (30.8)
Monotherapy, *N* (%)	35 (32.11)	11 (20.8)	44 (19.7)	33 (23.9)	47 (22.6)
Average number of ATH, mean (*SD*)	2.1 (1.0)	2.2 (0.8)	2.3 (1.0)	2.1 (0.9)	2.1 (0.8)
dCCBs, *N* (%)	70 (27.9)	89 (30.7)	133 (23.3)	74 (26.4)	185 (27.4)
Monotherapy, *N* (%)	11 (15.7)	44 (49.4)	34 (25.6)	24 (32.4)	88 (47.6)
Average number of ATH, mean (*SD*)	2.8 (1.2)	1.7 (0.8)	2.4 (1.1)	1.9 (0.8)	1.8 (0.9)

Abbreviations: ACE‐I, angiotensin converting enzyme inhibitor; ATH, antihypertensive; CARDIA, Coronary Artery Risk Development in Young Adult Study; CHS, Cardiovascular Health Study; dCCB, dihydropyridine calcium channel blocker; eGFR, estimated glomerular filtration rate; GENOA, Genetic Epidemiology Network of Atherosclerosis Study; HyperGEN, Hypertension Genetic Epidemiology Network; JHS, Jackson Heart Study; LVM, left ventricular mass; RWT, relative wall thickness; TD, thiazide diuretic.

### 3.2 Association between antihypertensive medication and LV traits

Results from the meta‐analyses of main effects (i.e., that of treatment on LV traits) are presented in Table 2 along with the sample sizes for each drug class comparison (ranging 721–1,001). Upon adjusting for covariates, the use of dCCBs was associated with higher LVM than the use of TDs (*p* = 0.001, effect size = 0.052 on the natural log scale (1.05 after back transformation)). The direction of effect was consistent across the five studies. ACE‐I in comparison to TD and dCCB in comparison to ACE‐I exposure were not associated with LVM or RWT.

**Table 2:** Meta‐analysis results of the main effect of antihypertensive medications on left ventricular traits among African Americans across five studies (*N* = 2,068)

Primary outcomes	Drug exposure								
	Model 1: ACE‐I versus TD (TD = ref) (*N* = 721)			Model 2: dCCB versus TD (TD = ref) (*N* = 926)			Model 3: dCCB versus ACE‐I (ACE‐I = ref) (*N* = 1,001)		
	β	*SE*	*p*‐value	β	*SE*	*p*‐value	β	*SE*	*p*‐value
LVM	0.030	0.018	0.104	0.052	0.016	**0.001**	0.017	0.016	0.292
RWT	−0.004	0.011	0.745	−0.003	0.011	0.770	0.016	0.010	0.104

Note

Fixed effect estimates were reported for all models. The bold value indicate the significant *P*‐value after multiple testing correction.

Abbreviations: ACE‐I, angiotensin converting enzyme inhibitor; dCCB, dihydropyridine calcium channel blocker; LVM, left ventricular mass; RWT, relative wall thickness; TD, thiazide diuretic.

Similar to LV traits, meta‐analysis of data from 935 AAs belonging to the CARDIA and HyperGEN studies was conducted for the secondary outcomes of *e*′ velocity, *E*/*e*′ ratio, and GLS (Table 3). dCCBs when compared with TDs were associated with higher lateral *E*/*e*′ (*p* = 0.006, effect size = 0.147 [1.16 after back transformation]). The direction of effect was positive in both studies. Similarly, dCCBs compared with TDs were associated with lower GLS (*p* = 0.042, effect size = −0.684).

**Table 3:** Meta‐analysis results of the main effect of antihypertensive medications on left ventricular functions among African Americans across CARDIA and HyperGEN studies (*N* = 935)

Secondary outcomes	Drug exposure								
	Model 1: ACE‐I versus TD (TD = ref) (*N* = 273)			Model 2: dCCB versus TD (TD = ref) (*N* = 349)			Model 3: dCCB versus ACE‐I (ACE‐I = ref) (*N* = 380)		
	β	*SE*	*p*‐value	β	*SE*	*p*‐value	β	*SE*	*p*‐value
Septal *e*′	−0.111	0.151	0.463	0.128	0.133	0.338	0.102	0.134	0.448
Lateral *e*′	−0.219	0.173	0.205	−0.197	0.148	0.183	0.008	0.151	0.959
Average *e*′	−0.136	0.149	0.360	−0.002	0.125	0.989	0.029	0.127	0.821
Septal *E*/*e*′	0.098	0.052	0.059	0.037	0.043	0.391	0.043	0.047	0.361
Lateral *E*/*e*′	0.119*	0.076*	0.118*	0.147*	0.053*	**0.006** *	0.048	0.053	0.371
Average *E*/*e*′	0.101*	0.052*	0.052*	0.081	0.045	0.072	0.045	0.046	0.335
GLS	−0.590	0.383	0.123	−0.684	0.336	**0.042**	0.274	0.318	0.389

Note

Fixed effect estimates were reported for all models except those with * (lateral *E*/*e*′ Model 1 and 2, and average *E*/*e*′ Model 1). The bold value indicates the significant *P*‐value after multiple testing correction.

Abbreviations: ACE‐I, angiotensin converting enzyme inhibitor; dCCB, dihydropyridine calcium channel blocker; GLS, global longitudinal strain; TD, thiazide diuretic.

### 3.3 The pharmacogenetic effect of antihypertensive medication on LV traits and functions

**Figure 1:**
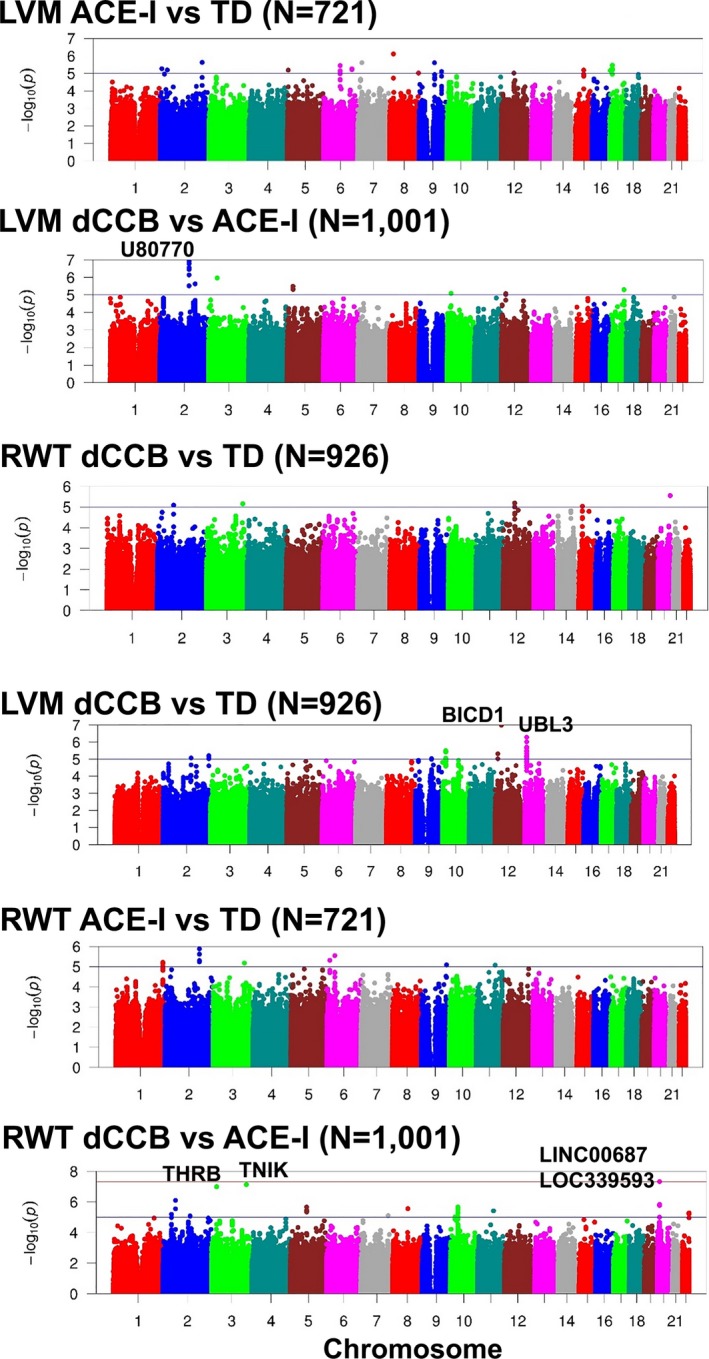
ACE‐I, angiotensin‐converting enzyme inhibitor. Within each chromosome, shown on the *x*‐axis, the results are plotted left to right from the pterminal end. The nearest genes are indicated for variants with an interaction *p*‐values less than 2 × 10^−6^ in the discovery meta‐analysis. Abbreviations: ACE‐I, angiotensin converting enzyme inhibitor; dCCB, dihydropyridine calcium channel blocker; LVM, left ventricular mass; RWT, relative wall thickness; TD, thiazide diuretic

**Figure 2:**
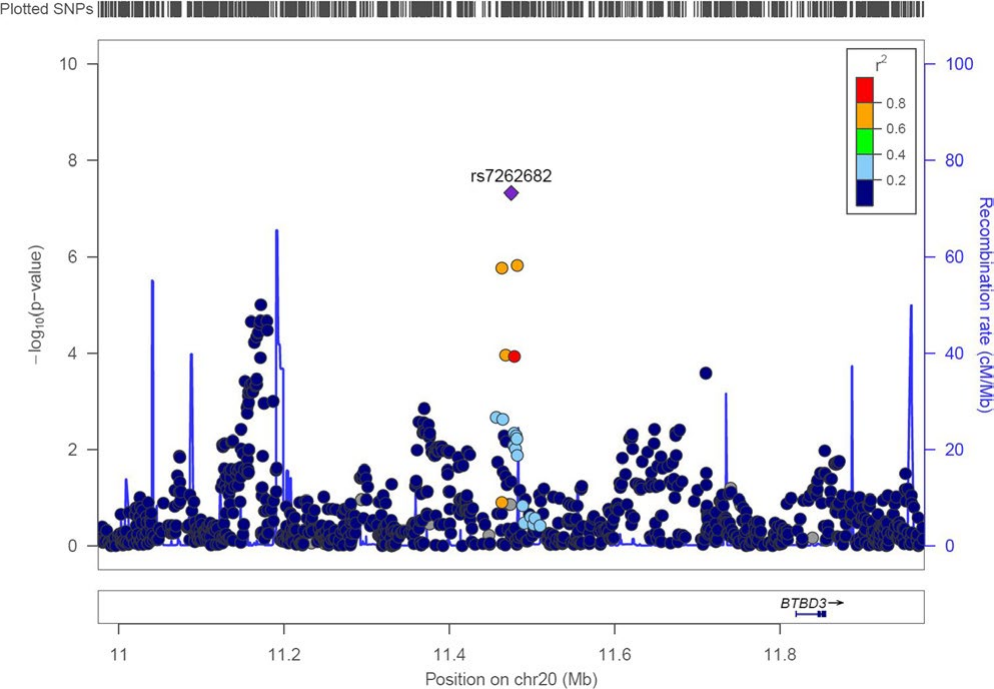
Locus zoom plot in region surrounding rs7262682 for relative wall thickness for dihydropyridine calcium channel blocker in comparison to angiotensin converting enzyme inhibitor

Q‐Q plots based on meta‐analyses for SNP‐by‐drug interaction parameters are presented in Figure S1. Variance inflation factors, λ, ranged from 0.978 to 1.021 (Table S2). We detected a genome‐wide significant SNP‐by‐drug interaction (*p* < 5 × 10^−8^) on RWT when comparing dCCBs to ACE‐Is observed for three SNPs within a 20 kb locus on chromosome 20. The directions of effect were consistent across four cohorts with available data (smallest *p* = 4.74 × 10^−8^) (see Table 4). The SNPs are located between long intergenic nonprotein coding RNA 687 (*LINC00687*) and long noncoding RNA (*LOC339593*) (Figures 1 and 2). All are common SNPs with MAF range of 0.09–0.12.

**Table 4:** Top interaction results of the meta‐analysis for LVM and RWT for three antihypertensive medication comparisons

RSID	Chr:BP	A1/A2	AF	Effect (*SE*)	Direction	*p*‐value	Location	Gene	Model
rs7262682	20:11,474,929	T/C	0.12	0.141 (0.026)	?++++	4.74 × 10^−8^	intergenic	*LINC00687*, *LOC339593*	RWT dCCB versus ACE
rs11906708	20:11,482,244	A/G	0.09	0.163 (0.034)	?++?+	1.48 × 10^−6^	Intergenic	*LINC00687*, *LOC339593*	RWT dCCB versus ACE
rs11906016	20:11,463,593	A/G	0.91	−0.159 (0.033)	?−−?−	1.71 × 10^−6^	Intergenic	*LINC00687*, *LOC339593*	RWT dCCB versus ACE
rs10176318	2:145,709,071	A/G	0.19	0.172 (0.033)	?++++	1.21 × 10^−7^	Intergenic	*U80770*	LVM dCCB versus ACE
rs7581822	2:145,697,961	A/G	0.82	−0.172 (0.033)	?−−−−	1.62 × 10^−7^	Intergenic	*U80770*	LVM dCCB versus ACE
rs13412736	2:145,714,109	T/C	0.20	0.162 (0.031)	−++++	1.66 × 10^−7^	Intergenic	*U80770*	LVM dCCB versus ACE
rs10193147	2:145,714,377	A/C	0.20	0.162 (0.031)	−++++	1.68 × 10^−7^	Intergenic	*U80770*	LVM dCCB versus ACE
rs10200130	2:145,706,040	T/C	0.20	0.158 (0.031)	−++++	2.78 × 10^−7^	Intergenic	*U80770*	LVM dCCB versus ACE
rs16824670	2:145,706,608	T/G	0.20	0.158 (0.031)	−++++	2.81 × 10^−7^	Intergenic	*U80770*	LVM dCCB versus ACE
rs10179372	2:145,709,987	A/C	0.20	0.157 (0.031)	−++++	3.47 × 10^−7^	Intergenic	*U80770*	LVM dCCB versus ACE
rs10204792	2:145,713,584	T/C	0.80	−0.157 (0.031)	+−−−−	3.54 × 10^−7^	Intergenic	*U80770*	LVM dCCB versus ACE
rs10496975	2:145,704,568	T/G	0.78	−0.152 (0.031)	+−−−−	7.28 × 10^−7^	Intergenic	*U80770*	LVM dCCB versus ACE
rs10172711	2:145,707,848	T/G	0.18	0.165 (0.035)	?++++	3.02 × 10^−6^	Intergenic	*U80770*	LVM dCCB versus ACE
rs9314972	13:29,225,969	A/G	0.55	−0.135 (0.027)	−−−−−	5.20 × 10^−7^	Intergenic	*UBL3*	LVM dCCB versus TD
rs9314974	13:29,230,437	T/C	0.40	0.127 (0.026)	+++++	9.67 × 10^−7^	Intergenic	*UBL3*	LVM dCCB versus TD
rs7995666	13:29,258,195	C/G	0.44	−0.126 (0.027)	−−−−−	1.95 × 10^−6^	Intronic	*UBL3*	LVM dCCB versus TD
rs9314973	13:29,230,070	T/G	0.37	−0.13 (0.027)	−−−−−	2.29 × 10^−6^	Intergenic	*UBL3*	LVM dCCB versus TD
rs1854176	13:29,231,262	T/G	0.46	0.122 (0.026)	+++++	3.16 × 10^−6^	Intergenic	*UBL3*	LVM dCCB versus TD
rs9551739	13:29,233,311	T/C	0.54	−0.121 (0.026)	−−−−−	3.56 × 10^−6^	Intergenic	*UBL3*	LVM dCCB versus TD
rs4769772	13:29,234,006	T/G	0.53	−0.12 (0.026)	−−−−−	4.61 × 10^−6^	Intergenic	*UBL3*	LVM dCCB versus TD
rs7330356	13:29,261,981	A/G	0.43	−0.118 (0.026)	−−−−−	6.15 × 10^−6^	Intronic	*UBL3*	LVM dCCB versus TD
rs9508554	13:29,258,860	T/C	0.45	0.118 (0.026)	+++++	6.53 × 10^−6^	Intronic	*UBL3*	LVM dCCB versus TD
rs1410110	13:29,263,017	T/C	0.45	0.118 (0.026)	+++++	7.05 × 10^−6^	Intronic	*UBL3*	LVM dCCB versus TD
rs326641	12:32,291,136	T/G	0.15	0.208 (0.039)	?++++	1.04 × 10^−7^	Intronic	*BICD1*	LVM dCCB versus TD
rs2217884	3:24,442,206	T/C	0.47	0.09 (0.017)	+++++	1.03 × 10^−7^	Intronic	*THRB*	RWT dCCB versus ACE

Note

Direction of effect by individual study were listed in order from left to right as CARDIA, CHS, HyperGEN, GENOA, and JHS.

Abbreviations: ACE‐I, angiotensin converting enzyme inhibitor; AF, allele frequency; BP, base‐pair position; CARDIA, Coronary Artery Risk Development in Young Adult Study; CHS, Cardiovascular Health Study; Chr, chromosome; dCCB, dihydropyridine calcium channel blocker; GENOA, Genetic Epidemiology Network of Atherosclerosis Study; HyperGEN, Hypertension Genetic Epidemiology Network; JHS, Jackson Heart Study; LVM, left ventricular mass; RSID, SNP identification; RWT, relative wall thickness; TD, thiazide diuretic.

Marginally significant SNP‐by‐drug findings included 10 SNPs near *U80770* on chromosome 2 for LVM when comparing dCCBs to ACE‐Is (smallest *p* = 1.21 × 10^−7^, MAF range of 0.17–0.22) (Figure 1). Ten SNPs near ubiquitin‐like 3 (*UBL3*) located on chromosome 13 modified the association with dCCBs in comparison to TD for LVM (smallest *p* = 5.20 × 10^−7^, MAF range of 0.37–0.47). *BICD1* rs326641 modified the association of dCCBs with LVM in comparison to TDs (*p* = 1.04 × 10^−7^, MAF = 0.15). *THRB* rs2217884 was associated with RWT when comparing dCCBs versus ACE‐Is (*p* = 1.03 × 10^−7^, MAF = 0.47). In a sensitivity analysis set in the HyperGEN AA cohort for the 25 SNPs identified in Table 4, we adjusted for dCCB use in the ACE‐I use versus TD use model, adjusted for ACE‐I use in the dCCB use versus TD use model, and adjusted for TD use in the dCCB use versus ACE‐I use model. The results are presented in the Table S3. The beta coefficients and *p*‐values for SNP‐by‐drug interaction of the 25 SNPs were consistent in the sensitivity analysis in comparison to original analyses in HyperGEN study (Table S4).

Similar to primary outcomes, GWAS analyses were performed using data from 935 AAs belonging to the CARDIA and HyperGEN studies for *e*′ velocity, *E*/*e*′ ratio, and GLS. Q‐Q plots based on meta‐analyses of the cohort‐specific, SNP‐by‐drug interaction parameters showed p‐values for the interaction terms followed expected trends with lambdas close to 1 for all models (Figure S2 and Table S5).

No genome‐wide significant interactions (*p* < 5 × 10^−8^) for any of the three drug comparisons on secondary outcomes were detected (Figure S3). However, we observed several marginally significant SNPs. Two SNPs (rs11744698 and rs6898102) near poly (ADP‐Ribose) polymerase family member 8 gene modified the association of dCCB in comparison to TD exposure for GLS (*p* = 7.59 × 10^−8^ and 8.18 × 10^−8^, respectively; both MAF = 0.29). We also found seven SNPs within 300 kb of protein phosphatase 2 regulatory subunit b‐alpha (*PPP2R3A*) which modified the association dCCB versus TD treatment with GLS (smallest *p* = 1.25 × 10^−7^, MAF range of 0.235–0.239). Fourteen SNPs within a 7 kb locus on chromosome 8 between ST3 beta‐galactoside alpha‐2,3‐sialyltransferase 1 and zinc finger and AT‐Hook domain containing (*ZFAT*) were associated with average *E*/*e*′ when comparing ACE‐Is to dCCBs (smallest *p* = 1.77 × 10^−7^, MAF range of 0.26–0.28). Finally, interactions between five intronic SNPs of coiled‐coil domain containing 3 and dCCB when compared with TD treatment were associated with septal *E*/*e*′ (smallest *p* = 2.7 × 10^−7^, MAF range of 0.16–0.21) (Table S6).

### 3.4 Validating the top results in the HyperGEN EAs

We sought to validate our findings from Table 4 in HyperGEN EAs. A total of 25 SNPs were identified in the discovery cohort (Table 4) plus 76 nearby SNPs (within 100 kb) in high linkage disequilibrium (*R* ^2^ ≥ 0.7) with those SNPs. Of those 101, 25 SNPs were found in the HyperGEN EA GWAS dataset with MAF ≥ 0.05 covering markers in *BICD1*, *THRB*, and *UBL3.* Results are presented in Table 5. The Bonferroni‐corrected threshold for significance was 0.002 (α = 0.05/25, where 25 is the number of SNPs for which we attempted replication). Two SNPs (rs326641 and rs326640) at *BICD1* modified the association of dCCBs with LVM in comparison to TDs (*p* = 0.0015 and 0.0019; MAF = 0.33 and 0.32, respectively). Rs326641 was observed in Table 4 and the direction of effect of the interaction term was consistent between the discovery cohorts and the validation cohort (+ for T vs. G). Two other SNPs (rs184469 and rs326639) at *BICD1* were marginally significant (*p* < 0.05) but did not pass the Bonferroni‐corrected significance threshold.

**Table 5:** Validating top interaction results of the meta‐analysis for LVM and RWT for three antihypertensive medication comparisons in the HyperGEN European Americans (*N* = 613)

RSID	Chr:BP	A1/A2	AF	Effect	*SE*	*p*‐value	Gene	Model
rs2217884	3:24,442,206	T/C	0.48	0.027	0.028	0.34	*THRB*	RWT dCCB versus ACE
rs13326381	3:24,447,577	C/T	0.43	−0.035	0.028	0.2	*THRB*	RWT dCCB versus ACE
rs4858613	3:24,442,912	G/A	0.42	−0.035	0.028	0.2	*THRB*	RWT dCCB versus ACE
rs326641	12:32,291,136	G/T	0.33	−0.140	0.044	0.0015	*BICD1*	LVM dCCB versus TD
rs326640	12:32,290,316	C/T	0.32	−0.138	0.044	0.0019	*BICD1*	LVM dCCB versus TD
rs184469	12:32,291,565	A/G	0.36	−0.086	0.028	0.0023	*BICD1*	LVM dCCB versus TD
rs326639	12:32,289,584	A/G	0.33	−0.109	0.047	0.02	*BICD1*	LVM dCCB versus TD
rs812645	12:32,290,865	T/G	0.41	0.078	0.044	0.078	*BICD1*	LVM dCCB versus TD
rs7330356	13:29,261,981	A/G	0.42	0.043	0.054	0.43	*UBL3*	LVM dCCB versus TD
rs9314972	13:29,225,969	A/G	0.34	0.043	0.054	0.43	*UBL3*	LVM dCCB versus TD
rs9551739	13:29,233,311	T/C	0.35	0.043	0.054	0.43	*UBL3*	LVM dCCB versus TD
rs1854176	13:29,231,262	G/T	0.35	0.043	0.054	0.43	*UBL3*	LVM dCCB versus TD
rs4769772	13:29,234,006	T/G	0.36	0.043	0.054	0.43	*UBL3*	LVM dCCB versus TD
rs1410110	13:29,263,017	C/T	0.36	0.043	0.054	0.43	*UBL3*	LVM dCCB versus TD
rs4265673	13:29,263,439	T/G	0.36	0.043	0.054	0.43	*UBL3*	LVM dCCB versus TD
rs7326253	13:29,273,270	T/C	0.36	−0.044	0.058	0.45	*UBL3*	LVM dCCB versus TD
rs9508554	13:29,258,860	C/T	0.36	0.043	0.054	0.43	*UBL3*	LVM dCCB versus TD
rs9578139	13:29,284,310	T/C	0.36	0.059	0.054	0.28	*UBL3*	LVM dCCB versus TD
rs9314974	13:29,230,437	C/T	0.33	0.043	0.054	0.43	*UBL3*	LVM dCCB versus TD
rs4238128	13:29,255,943	T/C	0.36	0.039	0.055	0.48	*UBL3*	LVM dCCB versus TD
rs7995666	13:29,258,195	C/G	0.40	0.043	0.054	0.43	*UBL3*	LVM dCCB versus TD
rs9578136	13:29,241,129	T/C	0.27	0.015	0.057	0.79	*UBL3*	LVM dCCB versus TD
rs2892463	13:29,245,835	T/G	0.42	0.000	0.055	0.99	*UBL3*	LVM dCCB versus TD
rs9314973	13:29,230,070	T/G	0.42	0.043	0.054	0.43	*UBL3*	LVM dCCB versus TD
rs957189	13:29,316,719	T/C	0.27	0.022	0.056	0.70	*UBL3*	LVM dCCB versus TD

Abbreviations: ACE‐I, angiotensin converting enzyme inhibitor; AF, allele frequency; BP, base‐pair position; Chr, chromosome; dCCB, dihydropyridine calcium channel blocker; HyperGEN, Hypertension Genetic Epidemiology Network; LVM, left ventricular mass; RSID, SNP identification; RWT, relative wall thickness; TD, thiazide diuretic.

## 4 DISCUSSION

In the present investigation, we combined samples from five observational epidemiological cohort studies to evaluate the association between antihypertensive medication class and LV traits as well as potential SNP‐by‐drug interactions among AAs treated for hypertension. We observed that dCCB use was associated with greater LVM than TD use. We also observed trends for poorer LV diastolic function when comparing dCCB to TD exposure, including lower GLS and higher *E*/*e*′ ratio. We observed one genome‐wide significant SNP‐by‐drug interaction effect on RWT among dCCB users in comparison to ACE‐I users. We also reported several marginally significant associations that provide preliminary evidence of SNP‐by‐drug interactions. Validation of study findings were attempted in an external EA cohort with comparable data.

We observed that dCCBs (compared with TDs) were associated with worse cardiac structure and function including higher LVM, higher lateral *E*/*e*′, and lower GLS. In an 80‐study meta‐analysis representing data on over 3,767 individuals treated for hypertension, Klingbeil et al. (2003) reported that LVM index decreased more with CCB treatment (average 11% decrease) compared to diuretic treatment (average 8% decrease). These results differ from ours, but Klingbeil et al. considered prospective changes in LVM index, did not stratify by ethnicity and did not restrict to the dCCB nor the TD subclasses. Similar to our findings, a randomized clinical trial of 53 hypertensive Japanese participants reported hydrochlorothiazide treatment in combination with angiotensin II receptor blocker treatment (ARB) was associated with greater improvement in LVM index in comparison to a dCCB/ARB treatment combination (Okura et al., 2013). Overall, these results highlight the importance of considering drug subclass in evaluating outcomes, and that TDs may be potentially associated with better LV structure and function in comparison to dCCBs in AAs treated for hypertension.

Our statistically significant SNP‐by‐drug interaction findings lie between two long noncoding RNA genes. Long noncoding RNAs regulate the expression of genes in the nucleus by directly interacting with DNA recruiting chromatin modifying complexes and various transcriptional regulators (Viereck & Thum, 2017). Additionally, they can be involved in epigenetic and transcriptional regulation of neighboring loci in *cis* or distal genes in *trans* (Viereck & Thum, 2017). Circulating levels of other long noncoding RNAs have been linked with acute heart failure, LV remodeling and other cardiovascular‐related outcomes (Viereck & Thum, 2017). SNPs in this locus were not available in our validation cohort though other studies have pointed to this region. A *LOC339593* variant, rs2207418 (a different variant than highlighted by our study) was associated with cardiac hypertrophy, heart failure, and mortality in a three‐stage analysis (Parsa et al., 2011). Specifically, rs2207418 was associated with hypertrophy among 1,610 unrelated Caucasian cases and 463 unrelated Caucasian controls (*p* = 8.9 × 10^–6^) in GWAS study (Parsa et al., 2011). The SNP was then associated with the increase of heart failure and mortality (RR = 1.85, *p* = 0.0019 and HR = 1.51, *p* = 4 × 10^–4^) among Caucasians in a follow‐up candidate gene study (Parsa et al., 2011). The results were validated in an Amish cohort in which the SNP was associated with the increase of LVM, heart failure risk, and heart failure mortality (Parsa et al., 2011). Another SNP on chromosome 20 rs77790871 (~500 kb away from the variant highlighted by our study) was associated with systolic blood pressure among European in GWAS study (Warren et al., 2017).

Other marginally significant loci for our primary outcomes include *U80770* which is not well characterized but is expressed in the heart and kidney. *BICD1* functions to affect telomere length in humans which is important for regulating DNA replication and cellular proliferation, and has been linked to aging (Mangino et al., 2008; Swift et al., 2010). Recently, *BICD1* was reported to directly modulate protease‐activated receptor‐1, a G protein‐coupled receptor that plays an important role in cardiomyocyte contractility (Swift et al., 2010). Previous studies reported main effect associations of *BICD1* variants with ejection fraction in EAs and with LVM among Caribbean Hispanics with high waist circumference (Della‐Morte et al., 2011; Huber et al., 2012). This result was validated in the HyperGEN EA population.

An interaction between *THRB* rs2217884 and dCCB versus ACE‐I treatment was weakly associated with RWT. The gene encodes the beta subunit of nuclear thyroid hormone receptor known to mediate the effect of its ligand on metabolism and heart rate (Pramfalk, Pedrelli, & Parini, 2011). Mutations in the gene reduce thyroid hormone signaling and cause a compensatory increase in T3 and T4 thyroid hormones. Additionally, higher circulating levels of T3 and/or T4 were correlated with higher LVM index and RWT among 293 hypertensive Japanese patients and 2078 middle‐aged EAs untreated for hypertension (Iida et al., 2012; Roef et al., 2013). Interestingly, daily use of nifedipine, a dCCB decreased T3 and T4 circulating levels on male albino rabbits during 3 months of treatment (Kaur, Mehta, Ambwani, & Gehlot, 2013). Additionally, thyroid hormone disorders can affect the synthesis and secretion of renin‐angiotensin system (RAS) components (Ichihara, Kobori, Miyashita, Hayashi, & Saruta, 1998; Santos & Ferreira, 2007; Vargas, Rodriguez‐Gomez, Vargas‐Tendero, Jimenez, & Montiel, 2012). ACE‐Is directly affects the RAS through blocking the conversion of angiotensin I to angiotensin II (Brown & Vaughan, 1998). Overall, there is biological plausibility for the SNP‐by‐drug interaction observed for RWT in the current study, and though the result was not validated in HyperGEN, future studies should continue to investigate if *THRB* variants modify the association of dCCBs versus ACE‐Is with RWT.

Among genes marginally associated with secondary outcomes (*e*′ velocity, *E*/*e*′ ratio and GLS), *PPP2R3A* encodes a regulatory subunit of the protein phosphatase 2 that is involved in negative control of cell growth and division. The gene is expressed in cardiomyocytes and has been associated with fibrinogen in a GWAS meta‐analysis of over 120,000 EAs (*p* = 2 × 10^−27^) and triglycerides in a GWAS meta‐analysis of over 62,000 EAs (*p* = 8 × 10^−9^) (de Vries et al., 2016). Another interesting gene is *ZFAT*, which encodes a zinc finger transcription factor involved in apoptosis and cell survival. Rare variants in *ZFAT* have been associated with hypertension in two different case‐control studies of EAs (Slavin, Feng, Schnell, Zhu, & Elston, 2011; Zhu, Feng, Li, Lu, & Elston, 2010).

Our study has several strengths. First, cardiac phenotypes were well‐measured using standardized methods of M‐mode echocardiography in all five cohorts. Second, our study focused on a specific subgroup of diuretics, TDs, and a specific subgroup of CCBs, dCCBs, obviating heterogeneous effects caused by different subclasses of antihypertensive medication with different mechanisms of actions.

Our study findings should also be interpreted in context of some limitations. First, this study used a cross‐sectional design that cannot establish temporality of the association between antihypertensive treatment and LV traits. Second, this study was designed to test for modest‐to‐large interaction effect sizes for common variants. Therefore, our study could not assess the associations of rare variants and other types of variants not well covered in our GWAS panels. Finally, we had secondary outcomes in only two of the five cohorts, limiting our statistical power to analyze these traits. Finally, we did not identify SNPs in the validation population for all the genes represented in Table 4 (*LINC00687/LOC339593*, *U80770*), therefore further validation of these findings is needed.

In this investigation, we report TDs are associated with better cardiac structure and function, and offer evidence supporting interactions between variants near/in *LINC00687*, *LOC339593*, *U80770*, *BICD1*, and* THRB* with antihypertensive medications on LV traits in AAs. Future studies are warranted to replicate the observed interactions in other populations of African descent, and sequencing studies in larger populations are needed to validate as well as deepen the resolution these findings. Importantly, this study suggests common variants could modify the association between antihypertensive treatment and LV traits in AAs, informing future precision medicine efforts in this population.

## CONFLICT OF INTEREST

The authors declared that they have no conflict of interest.
