# Supplementary material for: Genome‐wide meta‐analysis of SNP and antihypertensive medication interactions on left ventricular traits in African Americans
Source: Mol Genet Genomic Med. 2019 Aug 13;7(10):e00788. doi: 10.1002/mgg3.788 (PMC6785453; doi:10.1002/mgg3.788)
Supplement: Supplementary file 1 [file MGG3-7-e00788-s001.doc]

**SUPPLEMENTAL MATERIAL**

**Genome-wide Meta-analysis of SNP and Antihypertensive Medication Interactions on Left Ventricular Traits in African Americans**

**SUPPLEMENTAL METHODS**

**Discovery Populations and Phenotype Characterization and Harmonization**

**Discovery Populations**

***Coronary Artery Risk Development in Young Adult Study (CARDIA)***

CARDIA is a prospective study of risk factors for coronary heart disease in young adults from 18 to 30 years of age recruited during 1985-1986 (Friedman et al., 1988). It was conducted across four field centers including Birmingham, AL; Chicago, IL; Minneapolis, MN; and Oakland, CA (Friedman et al., 1988). Medical information including current treatment and history were ascertained through questionnaires (Spring et al., 2014). At baseline, participants brought in a list of all medications used (Spring et al., 2014) At l.ater exams, participants were asked to physically bring in their medications (Spring et al., 2014). Echocardiographic data and antihypertensive medication data was collected on ~2,000 African Americans (AAs) at year 25 (or exam 8) (Gardin, Wagenknecht, et al., 1995). Among them, 251 AAs with relevant drug exposures, GWAS data and echocardiography measurement at year 25 were included in this study.

***Cardiovascular Health Study (CHS)***

The CHS is a population-based cohort study of risk factors for CHD and stroke in adults age 65 years and older conducted across four field centers in the United States (Fried et al., 1991). The original predominately Caucasian cohort of 5,201 participants was recruited in 1989-1990 from random samples of the Medicare eligibility lists: subsequently, an additional predominately African-American cohort of 687 participants was enrolled for a total sample of 5,888 (Fried et al., 1991). Study participants were asked to bring all medications to the yearly in-person interview, and medication use was assessed at that time. Echocardiographic data were available on 510 AAs at visit 6, and antihypertensive treatment data was assessed at the same visit (Fox et al., 2013). Among these 510 participants, 290 AAs had relevant drug exposures, GWAS data, and echocardiography measurements, and were included in this study.

***Jackson Heart Study (JHS)***

JHS is a longitudinal, community-based cohort study from the Jackson, Mississippi area (Fuqua et al., 2005; Keku et al., 2005). The study aims to investigate risk factors and causes of CV diseases in adult AAs (21–84 years) (Fuqua et al., 2005). The study has an approximately 31% overlap with AAs from the Jackson, MS center for the ARIC study and includes a nested family study (Fuqua et al., 2005). Three exams were conducted in 2000-2004, 2005-2008, 2009-2012. During each clinical visit, participants presented all of the medications used within 2 weeks, including prescriptions, over the counter or herbal preparations (Harman et al., 2013). The Medi-Span® therapeutic classification system was used to identify medications. A registered pharmacist resolved and adjudicated any automated coding that led to indeterminate results. Echocardiography measurements and antihypertensive treatment data for 2,000 AAs and antihypertensive treatment data were collected at visit 1, excluding overlapping participants from ARIC (Fox et al., 2013). Among these, 571 AAs with relevant drug exposures, GWAS data and echocardiography measurement were included into this study; first degree relatives (n=8) were excluded to avoid inflation of test statistics.

***Genetic Epidemiology Network of Atherosclerosis study (GENOA)***

GENOA is a family-based study participating in the Family Blood Pressure Program (FBPP) (Investigators, 2002). GENOA consists of hypertensive sibships that were recruited for linkage and association studies in order to identify genes that influence blood pressure and its target organ damage. In the initial phase of the GENOA study (Phase I: 1996-2001), all members of sibships containing ≥ 2 individuals with essential hypertension clinically diagnosed before age 60 were invited to participate, including both hypertensive and normotensive siblings. GENOA participants include African Americans from Jackson, MS (N=1,854 at phase I) (Daniels et al., 2004). A second study visit was completed from 2001 to 2005 (Phase II) (Meyers, Chu, Mosley, & Kardia, 2010). During phase II, LV traits were measured on 1,090 African American participants. During the examinations at each phase, prescription medications taken by the participant during the previous month are recorded (Investigators, 2002). The Medi-Span® therapeutic classification system was used to identify medications (Daniels et al., 2004; Investigators, 2002). Each of prescription antihypertensive medications was assigned a 6-digit code number that categorizes antihypertensive medications into groups with similar modes of action (Investigators, 2002). A total of 280 AAs with relevant drug exposures, GWAS data and echocardiography measurement were included in this study.

***Hypertension Genetic Epidemiology Network study (HyperGEN)***

HyperGEN is a family based study with a sib-pair design. Hypertensive European American (EAs) and AAs sibships were recruited from population-based cohorts in Salt Lake City, UT, Minneapolis, MN, Forsyth County, NC, and from the community-at-large in Birmingham, AL, from 1995 to 2000 (Williams et al., 2000). Sibling pairs with onset of hypertension before age 60 were recruited in the first phase. The study was later extended to other siblings and the offspring of the hypertensive probands who were unmedicated adults (Investigators, 2002; Williams et al., 2000). Information on race, demographics, height, weight, current medications (via self-report) and co-morbid conditions as well as clinical measures (fasting serum chemistries and urine chemistries) was collected (Williams et al., 2000). Participants with type 1 diabetes or advanced renal disease (defined as serum creatinine level > 2 mg/dL) were excluded from the original study since these two conditions can cause secondary hypertension and the goal of HyperGEN was to identify novel essential hypertension loci (Williams et al., 2000). A total of 676 AAs treated for hypertension from HyperGEN had relevant data and were included in the discovery cohort. A total of 613 EAs treated for hypertension from HyperGEN served as the validation cohort.

**Phenotypic characterization and Harmonization**

***CARDIA***

At year 25, echocardiography was performed using an Artida cardiac ultrasound scanner (Toshiba Medical Systems, Otawara, Japan) (Appiah et al., 2016). LV traits were assessed by two-dimensional (2D) guided M-mode and Doppler echocardiography at all field centers by trained sonographers following a standardized protocol (Kishi et al., 2015). At the Johns Hopkins University reading center, experienced sonographers made measurements from digitized images using a standard software offline image‐analysis system (Kishi et al., 2015). Assessment of intra-reader and inter-reader agreement was performed during each study. LVM was calculated by using Devereux formula (Appiah et al., 2016). IVSDD, LVIDD, and PWTD were measured from 2-dimensional (2D)-guided M-mode echocardiograms obtained from optimized parasternal short-axis views (Appiah et al., 2016). Relative wall thickness (RWT) was calculated as twice the PWTD divided by the LVIDD.

Early peak diastolic mitral annular velocity (e') were measured from pulsed‐Doppler echocardiographic recordings of transmitral flow (Kishi et al., 2015). Using tissue Doppler imaging, e' was measured at the septal and lateral mitral annulus. Then, e’ was calculated from the average of the septal and lateral mitral annular velocities (Kishi et al., 2015).

***CHS***

At visit 6, all participants underwent two-dimensional (2D) guided M-mode echocardiography by trained sonographers following a standardized protocol (Gardin, Siscovick, et al., 1995). Videotapes were then displayed and digitalized at the echocardiography reading center at the University of California, Irvine (Gardin, Siscovick, et al., 1995). Next, LV measurements were made from digitized images using an offline image-analysis system equipped with customized computer algorithms (Gardin, Wagenknecht, et al., 1995). Quality control was performed at all center fields including standardized training of echo technicians and readers, periodic technician observation by a trained echocardiographer, blind duplicate readings to establish inter-reader and intra-reader measurement variabilities, periodic reader review sessions, phantom studies on the ultrasound equipment, and quality-control audits (Gardin, Wagenknecht, et al., 1995). The reproducibility of echocardiographic measurements was systematically assessed at visit six that yielded 17% mean difference and 14% median difference for inter-reader and 10% mean difference and 7% median difference for intra-reader of LV mass (Gardin, Siscovick, et al., 1995). Similar to CARDIA, LVM in CHS was calculated using Devereux formula. IVSDD, LVIDD, and PWTD were measured from 2-dimensional (2D)-guided M-mode echocardiograms (de Simone, Gottdiener, Chinali, & Maurer, 2008). Relative wall thickness (RWT) was calculated as the ratio of sum of the posterior wall thickness and interventricular septal thickness by the internal LV diameter (de Simone et al., 2008).

***JHS***

LV traits were measured by 2-dimensional (2D) transthoracic echocardiograms (Sonos-4500, Philips Medical Systems) using standardized protocols (Abdalla et al., 2016). A cardiologist performed all echocardiographic readings. Echocardiograms were then reviewed for clinical interpretation and analytical measurements by experienced cardiologists on networked image workstations (Abdalla et al., 2016). LVM in JHS was calculated by the Devereux formula similar to CARDIA and CHS. IVSDD, LVIDD, and PWTD were measured from 2-dimensional (2D) images from a parasternal short-axis view with the M-mode cursor positioned through the center of the ventricle (Abdalla et al., 2016; Fox et al., 2011). Relative wall thickness (RWT) was calculated as twice the PWTD divided by the LVIDD (Abdalla et al., 2016).

***GENOA***

LV traits were performed by two-dimensional (2D) guided M-mode and Doppler M-mode using an Acuson 128XP echo machine (Acuson, Mountain View, CA) (Fox et al., 2010). Readings were performed at the New York Presbyterian Hospital–Weill Cornell Medical Center and verified by a single highly experienced cardiologist following a standardized protocol (Fox et al., 2010). Correct orientation of planes for imaging and Doppler recordings was verified using standardized protocols (Meyers et al., 2010). Measurements were made using a computerized review station equipped with digitizing tablet and monitor screen overlay for calibration and performance of each measurement (Meyers et al., 2010). M-mode and 2D eachocardiograms via the parasternal acoustic window were used to recode LV structures for ≥10 beats (Meyers et al., 2010). LV measurements between separate echocardiograms are highly reliable (e.g., the correlation between repeated measures of LVM was 0.93 between paired echocardiograms in hypertensive adults) (Meyers et al., 2010). Similar to other studies, LVM was calculated by Devereux formula (Devereux, Casale, Eisenberg, Miller, & Kligfield, 1984) (Arnett et al., 2011). IVSDD, LVIDD, and PWTD were measured by M-mode or 2D echocardiography (Arnett et al., 2011). RWT was calculated as twice the PWTD divided by the LVIDD (Arnett et al., 2011).

***HyperGEN***

LV traits were assessed by two-dimensional (2D) guided M-mode and Doppler echocardiography at the Salt Lake City, Minneapolis, Birmingham, and Forsyth County field centers following a standardized protocol. All instruments were calibrated against a standard phantom at installation and were validated regularly (Tang et al., 2002). Certificated sonographers from each center were also trained at the echocardiography reading center at New York Hospital-Weill Cornell Medical Center. At the reading center, measurements were computerized, calibrated, and quantified using a review station with digitizing tablet and monitor overlay (Arnett et al., 2009). LV measurements between separate echocardiograms by the reading center were reliable (e.g., intra class correlation coefficient is 0.93 for LV mass) (Tang et al., 2002).

Similar to other studies, LVM was calculated by Devereux formula.(Arnett et al., 2011; Devereux et al., 1984) IVSDD, LVIDD, and PWTD were measured by M-mode or 2D echocardiography (Arnett et al., 2011). RWT was calculated as twice the PWTD divided by the LVIDD (Arnett et al., 2011).

**LIST OF MEDICATION INVENTORY AND MEDICATION EXCLUSIONS FOR INDIVIDUAL MODEL**


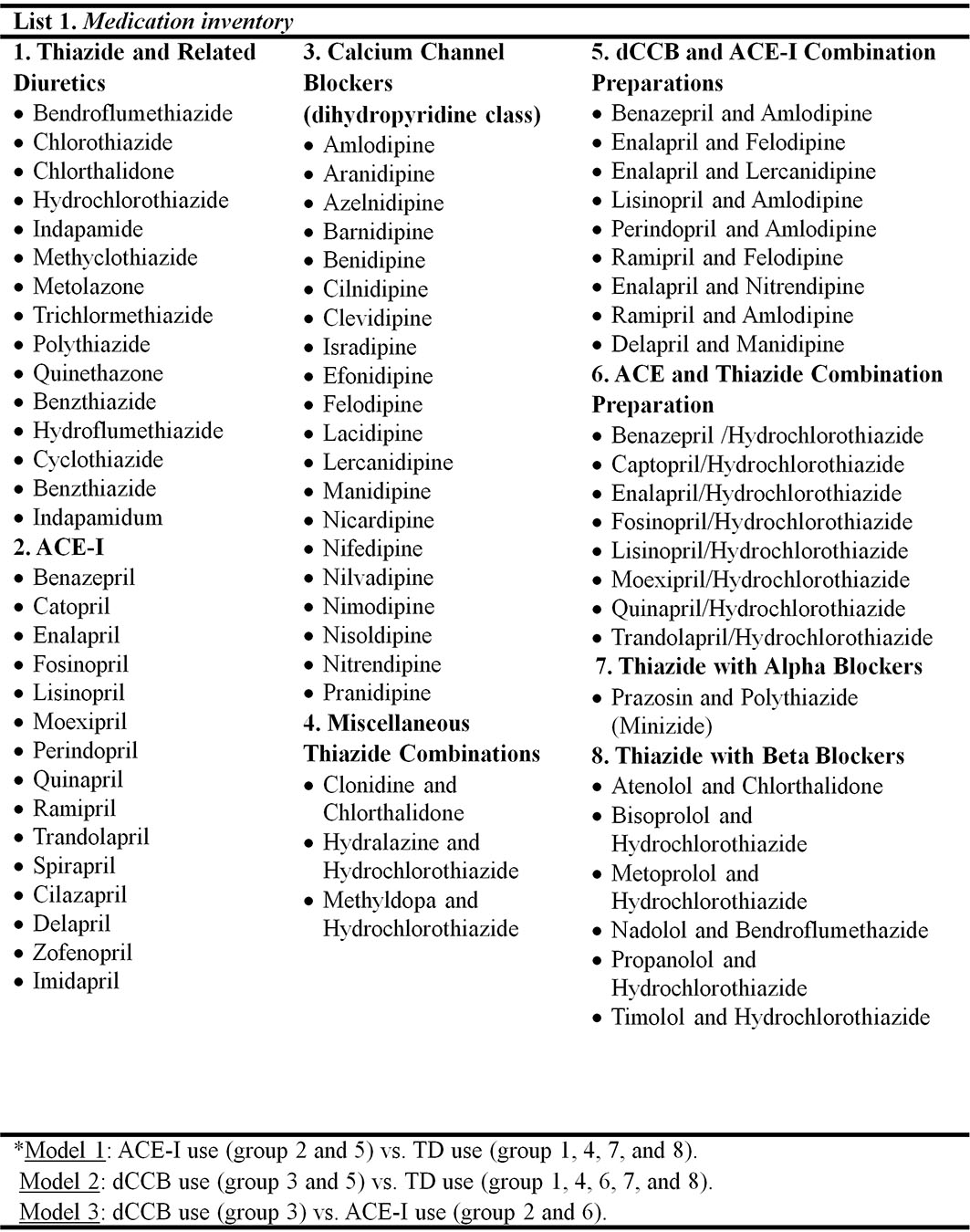


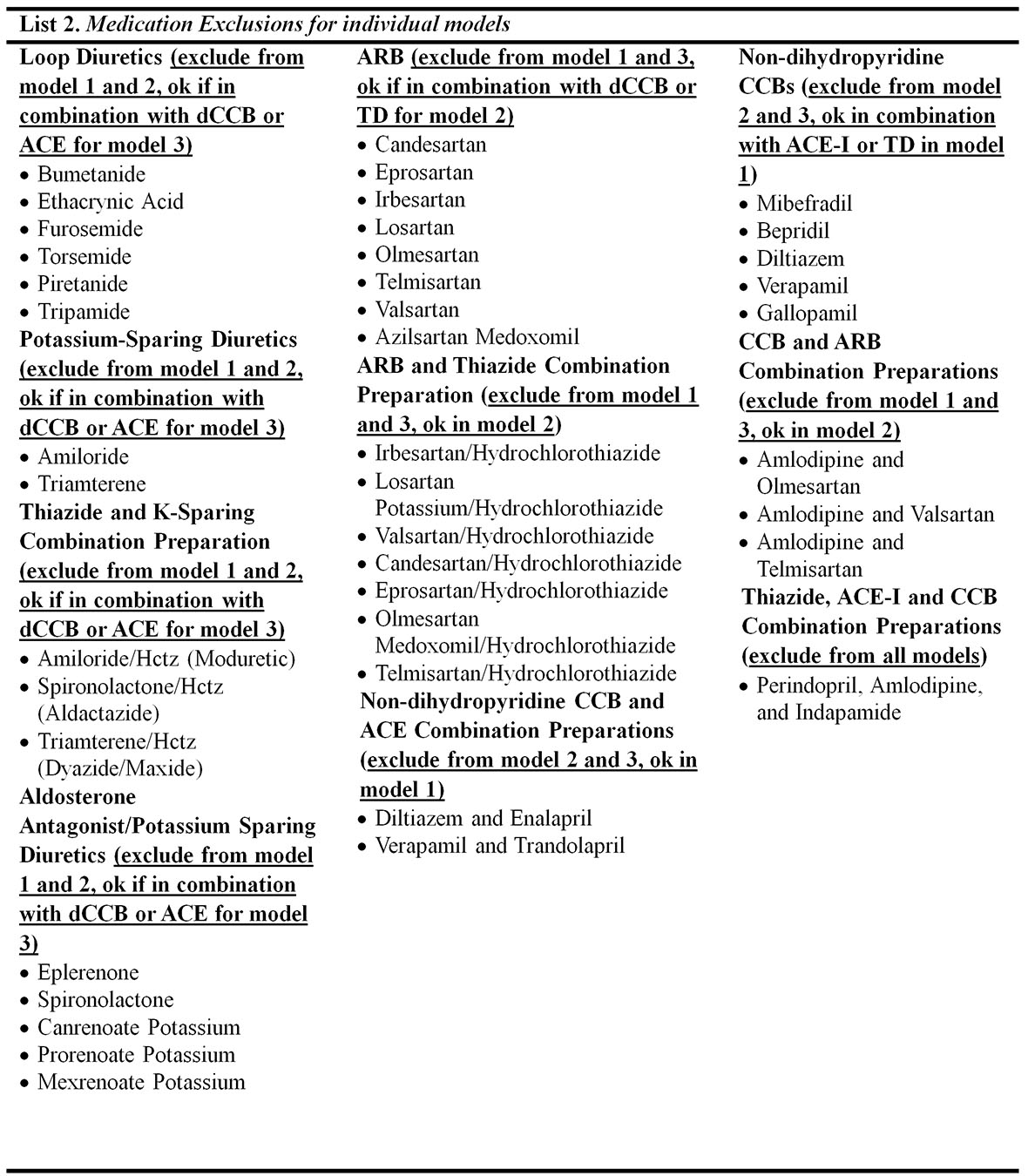


**SUPPLEMENTAL RESULTS**


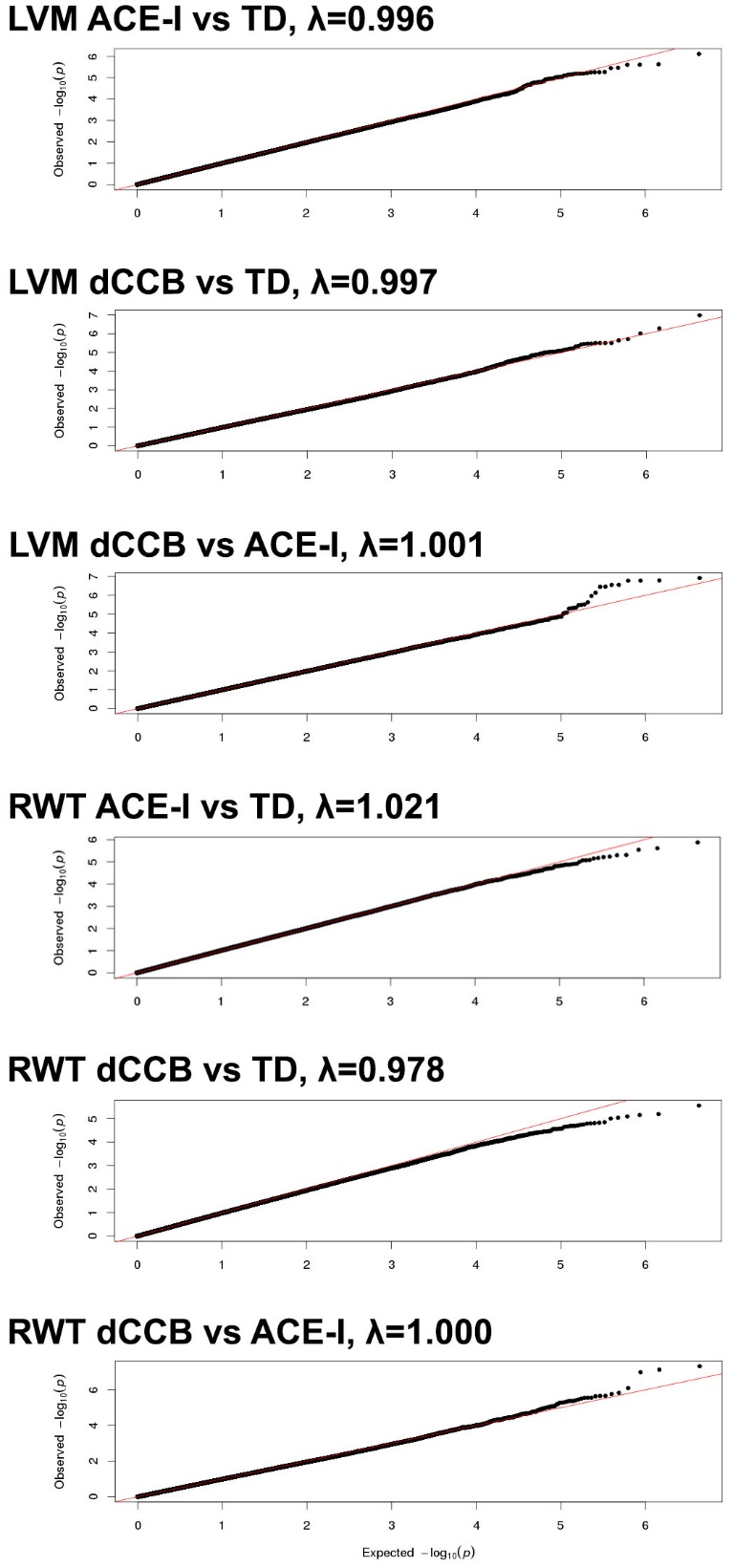


**FIGURE S1** **Q-Q plots for discovery meta-analyses for left ventricular mass (LVM) and relative wall thickness (RWT) for three anti-hypertensive medication comparisons**. ACE-I, angiotensin-coverting enzyme inhibitor; dCCB, dihydropyridine calcium channel blocker; TD, thiazide diuretic; λ indicates the genomic inflation factor.


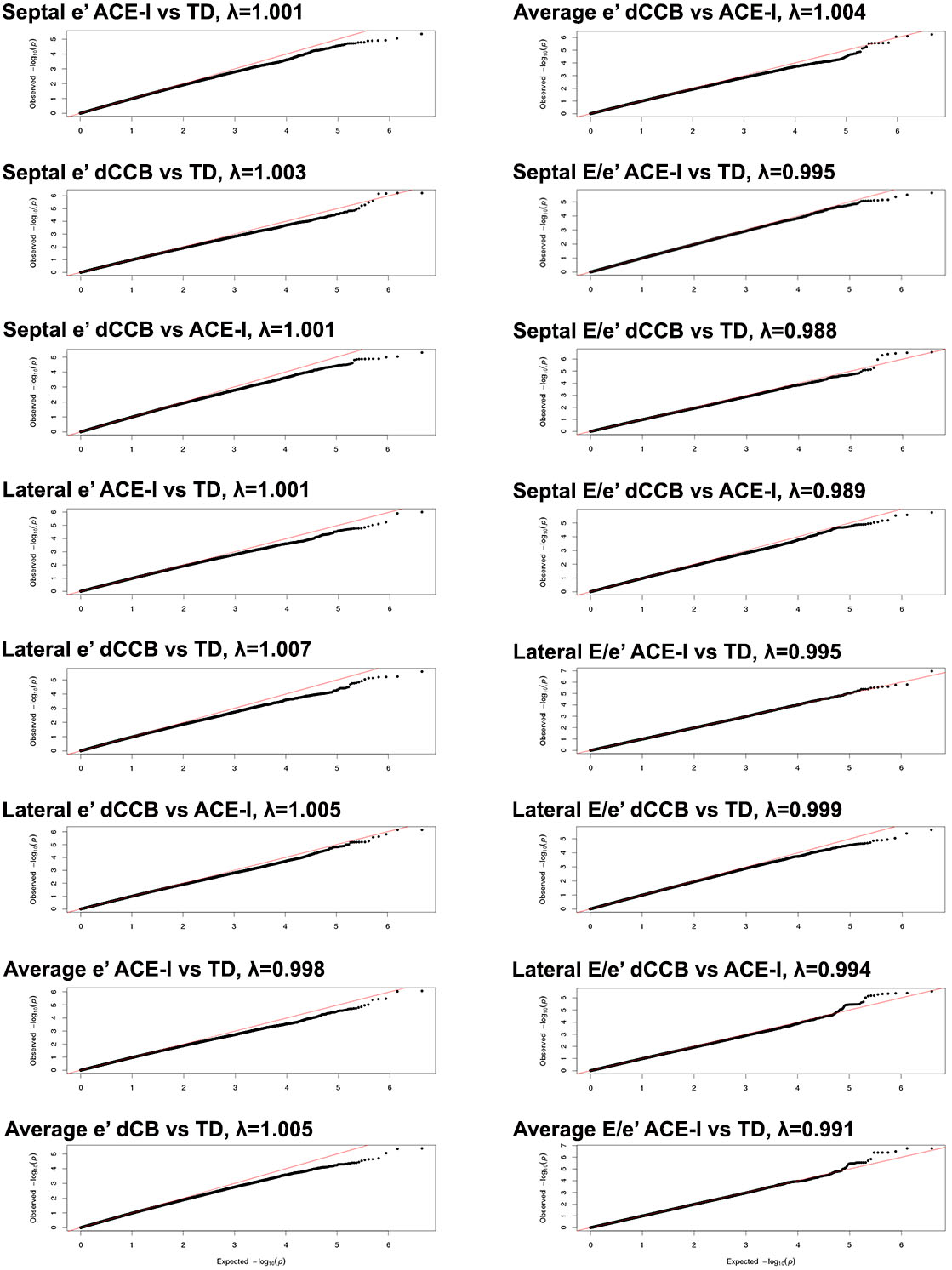
/

**FIGURE S2** **Q-Q plots for discovery meta-analyses for left ventricular function for three anti-hypertensive medication comparisons.** ACE-I, angiotensin-coverting enzyme inhibitor; dCCB, dihydropyridine calcium channel blocker; TD, thiazide diuretic; λ indicates genomic inflation factor; GLS, global longitudinal strain.


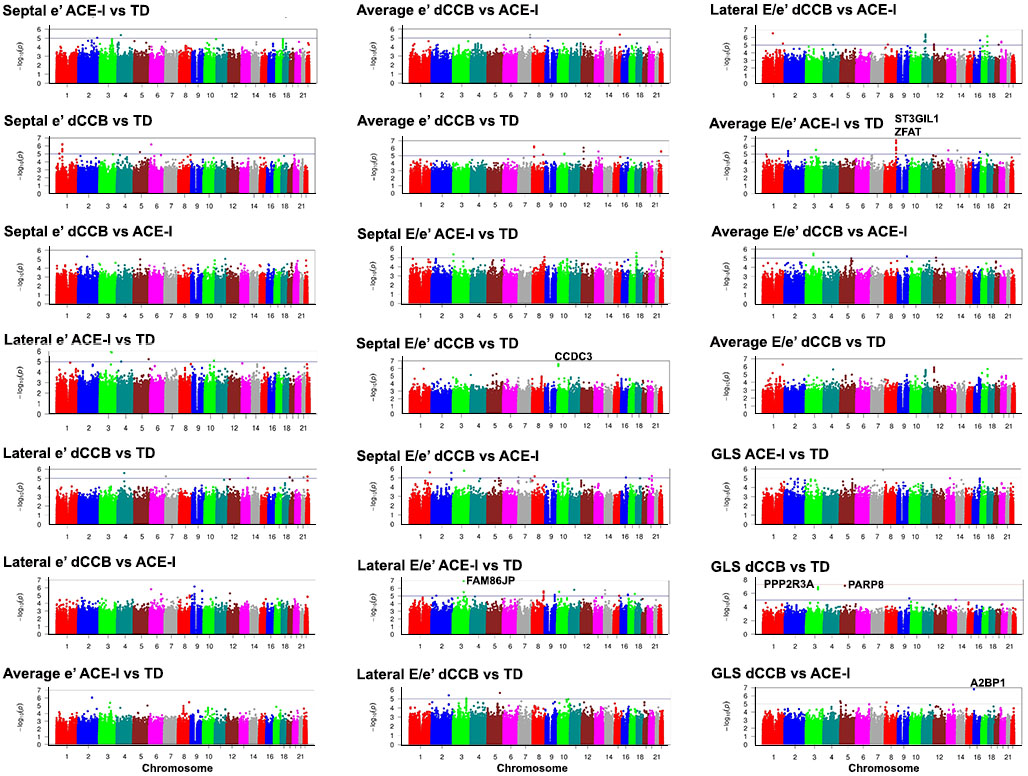


**FIGURE S3** **Plots show the individual interaction p-values based on discovery meta-analysis against their genomic position for left ventricular function for three anti-hypertensive medication comparisons.** ACE-I, angiotensin-coverting enzyme inhibitor; dCCB, dihydropyridine calcium channel blocker; TD, thiazide diuretic; GLS, global longitudinal strain. Within each chromosome, shown on the x-axis, the results are plotted left to right from the p-terminal end. The nearest genes are indicated for variants with an interaction p-values less than 3*10^-7^ in the discovery meta-analysis.

| **TABLE S1 Genotype description by cohorts** | | | | | |
| --- | --- | --- | --- | --- | --- |
|  | **CARDIA** | **CHS** | **JHS** | **GENOA** | **HyperGEN** |
| **Collection type** | Community-based | Community-based | Community and family based | Family-based | Family-based |
| **Special exclusions** |  |  |  | PC outliers | Participants with type 1 diabetes or advanced renal disease (defined as serum creatinine level > 2 mg/dL) were excluded from the original study since these two conditions can cause secondary hypertension and the goal of HyperGEN was to identify novel essential hypertension loci. |
| **Genotyping platform and SNP panel** | Affymetrix 6.0 | Illumina Human1M-Duo | Affymetrix 6.0 | 83% with the Affymetrix 6.0 and 17% with Illumina Human1M and 660-Quad | 86% with the Affy 6.0 and 14% with Affy 5.0 |
| **Genotyping calling algorithm** | Plink/ Birdseed | Illumina GenomeStudio | Birdseed | Birdseed, Illumina GenomeStudio | Birdseed |
| **Call rate (filter detail / N individuals excluded)** | <95% | < 95% | < 95% | <95% | 95% |
| **Other sample QC** | Sex discrepancy, duplicate discordance, cryptic relatedness | Sex discrepancy Discordant with prior genotyping | Sample duplicates, contaminated samples, excess heterozygosity, cryptic relatedness, sample outliers | Sex discrepancy, duplicate samples, samples with low identity-by-state | contaminated samples, blood sample mix-ups |
| **SNP call freq** | < 90% | < 97% | < 90% | < 95% | < 95% |
| **Other SNP QC filters applied?** | MAF<1%, HWE p <10^-6^, mapping to several genomic locations | HWE P < 10^−5^ > 2 DE/MeI hz frequency = 0, not in HapMap | MAF < 0.01 HWE p < 10^−6^, MeI , mapping to several genomic locations | MAF<1% | mendelian errors, MAF <1%, HWE P<10^-6^ |
| **SNP number in QC'd dataset** | 855,408 | 940,567 | 868,969 | Illumina: 1047639  Affymetrix: 762766" | 846,813 |
| **Imputation software** | MACH 1.0.16 | MACH | MACH | MACH | MACH 1.0.16 |
| **Imputation reference panel** | HapMap P2.r22.b36, 1:1 CEU:YRI | HapMap P2.r22.b36, (CEU, YRI) & HapMap Phase 3 (YRI, ASW, CEU) | HapMap P2.r22.b36, CEU+YRI | HapMap P2.r22.b36, (CEU, YRI) | HapMap 2, CEU and YRI |
| **Imputation quality filters** | rsq>0.5 and MAF > 1.0% | effective allele count(EAC) >10, EAC=allele count*r^2^ imputation quality | rsq>0.3 | none | r2hat > 0.3 |
| **Adjustments** | age, sex, height, weight, t2d status, number of anti-hypertensive treatment classes, eGFR, 4PCs | age, sex, height, weight, t2d status, number of anti-hypertensive treatment classes, eGFR, 10PCs | age, sex, weight, height, number of hypertension medications, diabetes, eGFR (CKD-EPI equation), 10 PCs | age, sex, height, weight, eGFR, diabetes, count of antihypertensive treatment classes, 10PCs | age, sex, center, height, weight, count of antihypertensive treatment classes, type 2 diabetes, eGFR, 10PCs |
| **Analysis method** | Linear regression | Linear regression | Generalized estimation equation | Generalized estimation equation | Linear mixed effect models |
| **Familial relatedness adjustment** | NA | NA | First degree relatives excluded | Family information as a matrix | Family information as a matrix |
| **Software for analysis** | ProABEL | R | ProbAbel | geepack r package (geeglm()) | LMEKIN |

| **TABLE S2 Number of SNPs and genomic inflation factors for discovery meta-analyses of left ventricular traits** | | | |
| --- | --- | --- | --- |
| **Outcome** | **Model** | **Number of SNPs** | **lambda** |
| **LVM** | Model 1: ACE-I vs. TD (TD=ref) | 2,158,834 | 0.996 |
| **LVM** | Model 2: dCCB vs. TD (TD=ref) | 2,131,686 | 0.997 |
| **LVM** | Model 3: dCCB vs. ACE-I (ACE-I=ref) | 2,191,657 | 1.001 |
| **RWT** | Model 1: ACE-I vs. TD (TD=ref) | 2,159,038 | 1.021 |
| **RWT** | Model 2: dCCB vs. TD (TD=ref) | 2,131,688 | 0.978 |
| **RWT** | Model 3: dCCB vs. ACE-I (ACE-I=ref) | 2,191,661 | 1.000 |
| Table shows the number of SNPs available for analysis after QC parameters were applied at the level of the individual studies and across all studies contributing to the discovery meta-analysis. Lambda indicates the genomic inflation factor. LVM, left ventricular mass; RWT, relative wall thickness; ACE-I, angiotensin-converting enzyme inhibitor; dCCB, dihydropyridine calcium channel blocker; TD, thiazide diuretic. | | | |

| **SUPPLEMENTAL TABLE 3 Sensitivity analysis results of top 25 SNPs identified in the discovery cohort for left ventricular mass and relative wall thickness for three antihypertensive medication comparisons in HyperGEN** | | | | | | | | |
| --- | --- | --- | --- | --- | --- | --- | --- | --- |
| **RSID** | **Chr:BP** | **A1/A2** | **AF** | **Interaction Effect (SE)** | **P-value** | **Location** | **Gene** | **Model** |
| rs7262682 | 20:11474929 | T/C | 0.12 | 0.107 (0.035) | 0.002535 | intergenic | *LINC00687, LOC339593* | RWT dCCB vs ACE |
| rs11906708 | 20:11482244 | A/G | 0.09 | 0.128 (0.04) | 0.001446 | intergenic | *LINC00687, LOC339593* | RWT dCCB vs ACE |
| rs11906016 | 20:11463593 | A/G | 0.91 | 0.12 (0.04) | 0.002386 | intergenic | *LINC00687, LOC339593* | RWT dCCB vs ACE |
| rs10176318 | 2:145709071 | A/G | 0.19 | 0.129 (0.045) | 0.004192 | intergenic | *U80770* | LVM dCCB vs ACE |
| rs7581822 | 2:145697961 | A/G | 0.82 | 0.129 (0.045) | 0.004192 | intergenic | *U80770* | LVM dCCB vs ACE |
| rs13412736 | 2:145714109 | T/C | 0.20 | 0.136 (0.045) | 0.002495 | intergenic | *U80770* | LVM dCCB vs ACE |
| rs10193147 | 2:145714377 | A/C | 0.20 | 0.136 (0.045) | 0.002495 | intergenic | *U80770* | LVM dCCB vs ACE |
| rs10200130 | 2:145706040 | T/C | 0.20 | 0.126 (0.045) | 0.005203 | intergenic | *U80770* | LVM dCCB vs ACE |
| rs16824670 | 2:145706608 | T/G | 0.20 | 0.126 (0.045) | 0.005203 | intergenic | *U80770* | LVM dCCB vs ACE |
| rs10179372 | 2:145709987 | A/C | 0.20 | 0.122 (0.045) | 0.006642 | intergenic | *U80770* | LVM dCCB vs ACE |
| rs10204792 | 2:145713584 | T/C | 0.80 | 0.122 (0.045) | 0.006642 | intergenic | *U80770* | LVM dCCB vs ACE |
| rs10496975 | 2:145704568 | T/G | 0.78 | 0.127 (0.045) | 0.004705 | intergenic | *U80770* | LVM dCCB vs ACE |
| rs10172711 | 2:145707848 | T/G | 0.18 | 0.115 (0.048) | 0.016976 | intergenic | *U80770* | LVM dCCB vs ACE |
| rs9314972 | 13:29225969 | A/G | 0.55 | 0.14 (0.037) | 0.000124 | intergenic | *UBL3* | LVM dCCB vs TD |
| rs9314974 | 13:29230437 | T/C | 0.40 | 0.126 (0.038) | 0.000786 | intergenic | *UBL3* | LVM dCCB vs TD |
| rs7995666 | 13:29258195 | C/G | 0.44 | 0.161 (0.037) | 1.18E-05 | intronic | *UBL3* | LVM dCCB vs TD |
| rs9314973 | 13:29230070 | T/G | 0.37 | 0.143 (0.037) | 0.00012 | intergenic | *UBL3* | LVM dCCB vs TD |
| rs1854176 | 13:29231262 | T/G | 0.46 | 0.132 (0.036) | 0.000266 | intergenic | *UBL3* | LVM dCCB vs TD |
| rs9551739 | 13:29233311 | T/C | 0.54 | 0.132 (0.036) | 0.000266 | intergenic | *UBL3* | LVM dCCB vs TD |
| rs4769772 | 13:29234006 | T/G | 0.53 | 0.129 (0.037) | 0.000447 | intergenic | *UBL3* | LVM dCCB vs TD |
| rs7330356 | 13:29261981 | A/G | 0.43 | 0.14 (0.038) | 0.00023 | intronic | *UBL3* | LVM dCCB vs TD |
| rs9508554 | 13:29258860 | T/C | 0.45 | 0.15 (0.036) | 3.73E-05 | intronic | *UBL3* | LVM dCCB vs TD |
| rs1410110 | 13:29263017 | T/C | 0.45 | 0.15 (0.036) | 3.73E-05 | intronic | *UBL3* | LVM dCCB vs TD |
| rs326641 | 12:32291136 | T/G | 0.15 | -0.217 (0.052) | 3.31E-05 | intronic | *BICD1* | LVM dCCB vs TD |
| rs2217884 | 3:24442206 | T/C | 0.47 | -0.11 (0.022) | 5.93E-07 | intronic | *THRB* | RWT dCCB vs ACE |
| RSID, SNP identification; AF, allele frequency; BP, base-pair position; Chr, chromosome; SE, standard error; LVM, left ventricular mass; RWT, relative wall thickness; ACE-I, angiotensin-converting enzyme inhibitor; dCCB, dihydropyridine calcium channel blocker; TD, thiazide diuretic. Models were adjusted for age, sex, weight, height, other drug, estimated glomerular filtration rate (eGFR), and type 2 diabetes (T2D) | | | | | | | | |

| **SUPPLEMENTAL TABLE 4 Results of top 25 SNPs identified in the discovery cohort for left ventricular mass and relative wall thickness for three antihypertensive medication comparisons in HyperGEN** | | | | | | | | |
| --- | --- | --- | --- | --- | --- | --- | --- | --- |
| **RSID** | **Chr:BP** | **A1/A2** | **AF** | **Interaction Effect (SE)** | **P-value** | **Location** | **Gene** | **Model** |
| rs7262682 | 20:11474929 | T/C | 0.12 | 0.111 (0.037) | 0.003063 | intergenic | *LINC00687, LOC339593* | RWT dCCB vs ACE |
| rs11906708 | 20:11482244 | A/G | 0.09 | 0.132 (0.042) | 0.00203 | intergenic | *LINC00687, LOC339593* | RWT dCCB vs ACE |
| rs11906016 | 20:11463593 | A/G | 0.91 | 0.124 (0.042) | 0.003227 | intergenic | *LINC00687, LOC339593* | RWT dCCB vs ACE |
| rs10176318 | 2:145709071 | A/G | 0.19 | 0.139 (0.047) | 0.003776 | intergenic | *U80770* | LVM dCCB vs ACE |
| rs7581822 | 2:145697961 | A/G | 0.82 | 0.139 (0.047) | 0.003776 | intergenic | *U80770* | LVM dCCB vs ACE |
| rs13412736 | 2:145714109 | T/C | 0.20 | 0.145 (0.047) | 0.002365 | intergenic | *U80770* | LVM dCCB vs ACE |
| rs10193147 | 2:145714377 | A/C | 0.20 | 0.145 (0.047) | 0.002365 | intergenic | *U80770* | LVM dCCB vs ACE |
| rs10200130 | 2:145706040 | T/C | 0.20 | 0.135 (0.047) | 0.004643 | intergenic | *U80770* | LVM dCCB vs ACE |
| rs16824670 | 2:145706608 | T/G | 0.20 | 0.135 (0.047) | 0.004643 | intergenic | *U80770* | LVM dCCB vs ACE |
| rs10179372 | 2:145709987 | A/C | 0.20 | 0.132 (0.047) | 0.005578 | intergenic | *U80770* | LVM dCCB vs ACE |
| rs10204792 | 2:145713584 | T/C | 0.80 | 0.132 (0.047) | 0.005578 | intergenic | *U80770* | LVM dCCB vs ACE |
| rs10496975 | 2:145704568 | T/G | 0.78 | 0.136 (0.047) | 0.004279 | intergenic | *U80770* | LVM dCCB vs ACE |
| rs10172711 | 2:145707848 | T/G | 0.18 | 0.119 (0.051) | 0.01938 | intergenic | *U80770* | LVM dCCB vs ACE |
| rs9314972 | 13:29225969 | A/G | 0.55 | 0.143 (0.038) | 0.000246 | intergenic | *UBL3* | LVM dCCB vs TD |
| rs9314974 | 13:29230437 | T/C | 0.40 | 0.128 (0.039) | 0.001407 | intergenic | *UBL3* | LVM dCCB vs TD |
| rs7995666 | 13:29258195 | C/G | 0.44 | 0.164 (0.038) | 3.15E-05 | intronic | *UBL3* | LVM dCCB vs TD |
| rs9314973 | 13:29230070 | T/G | 0.37 | 0.145 (0.039) | 0.000255 | intergenic | *UBL3* | LVM dCCB vs TD |
| rs1854176 | 13:29231262 | T/G | 0.46 | 0.134 (0.038) | 0.000516 | intergenic | *UBL3* | LVM dCCB vs TD |
| rs9551739 | 13:29233311 | T/C | 0.54 | 0.134 (0.038) | 0.000516 | intergenic | *UBL3* | LVM dCCB vs TD |
| rs4769772 | 13:29234006 | T/G | 0.53 | 0.131 (0.039) | 0.000836 | intergenic | *UBL3* | LVM dCCB vs TD |
| rs7330356 | 13:29261981 | A/G | 0.43 | 0.142 (0.04) | 0.000465 | intronic | *UBL3* | LVM dCCB vs TD |
| rs9508554 | 13:29258860 | T/C | 0.45 | 0.151 (0.038) | 9.98E-05 | intronic | *UBL3* | LVM dCCB vs TD |
| rs1410110 | 13:29263017 | T/C | 0.45 | 0.151 (0.038) | 9.98E-05 | intronic | *UBL3* | LVM dCCB vs TD |
| rs326641 | 12:32291136 | T/G | 0.15 | -0.219 (0.055) | 8.71E-05 | intronic | *BICD1* | LVM dCCB vs TD |
| rs2217884 | 3:24442206 | T/C | 0.47 | -0.108 (0.023) | 5.34E-06 | intronic | *THRB* | RWT dCCB vs ACE |
| RSID, SNP identification; AF, allele frequency; BP, base-pair position; Chr, chromosome; SE, standard error; LVM, left ventricular mass; RWT, relative wall thickness; ACE-I, angiotensin-converting enzyme inhibitor; dCCB, dihydropyridine calcium channel blocker; TD, thiazide diuretic. Models were adjusted for age, sex, weight, height, count of antihypertensive treatment classes, estimated glomerular filtration rate (eGFR), and type 2 diabetes (T2D) | | | | | | | | |

| **TABLE S5 Number of SNPs and genomic inflation factors for discovery meta-analyses of left ventricular functions** | | | |
| --- | --- | --- | --- |
| **Outcome** | **Model** | **Number of SNPs** | **lambda** |
| **septal e’** | Model 1: ACE-I vs. TD (TD=ref) | 1,856,615 | 1.001 |
| **septal e’** | Model 2: dCCB vs. TD (TD=ref) | 1,532,209 | 1.003 |
| **septal e’** | Model 3: dCCB vs. ACE-I (ACE-I=ref) | 1,541,650 | 1.001 |
| **lateral e’** | Model 1: ACE-I vs. TD (TD=ref) | 1,866,188 | 1.001 |
| **lateral e’** | Model 2: dCCB vs. TD (TD=ref) | 1,528,944 | 1.007 |
| **lateral e’** | Model 3: dCCB vs. ACE-I (ACE-I=ref) | 1,536,564 | 1.005 |
| **average e’** | Model 1: ACE-I vs. TD (TD=ref) | 1,855,715 | 0.998 |
| **average e’** | Model 2: dCCB vs. TD (TD=ref) | 1,529,874 | 1.005 |
| **average e’** | Model 3: dCCB vs. ACE-I (ACE-I=ref) | 1,541,592 | 1.004 |
| **septal E/e’** | Model 1: ACE-I vs. TD (TD=ref) | 1,455,135 | 0.995 |
| **septal E/e’** | Model 2: dCCB vs. TD (TD=ref) | 1,524,051 | 0.988 |
| **septal E/e’** | Model 3: dCCB vs. ACE-I (ACE-I=ref) | 1,527,316 | 0.989 |
| **lateral E/e’** | Model 1: ACE-I vs. TD (TD=ref) | 1,455,346 | 0.995 |
| **lateral E/e’** | Model 2: dCCB vs. TD (TD=ref) | 1,524,246 | 0.999 |
| **lateral E/e’** | Model 3: dCCB vs. ACE-I (ACE-I=ref) | 1,538,186 | 0.994 |
| **average E/e’** | Model 1: ACE-I vs. TD (TD=ref) | 1,455,201 | 0.991 |
| **average E/e’** | Model 2: dCCB vs. TD (TD=ref) | 1,523,486 | 0.983 |
| **average E/e’** | Model 3: dCCB vs. ACE-I (ACE-I=ref) | 1,529,948 | 0.994 |
| **GLS** | Model 1: ACE-I vs. TD (TD=ref) | 1,777,048 | 0.990 |
| **GLS** | Model 2: dCCB vs. TD (TD=ref) | 1,389,060 | 0.996 |
| **GLS** | Model 3: dCCB vs. ACE-I (ACE-I=ref) | 1,439,601 | 0.998 |
| Table shows the number of SNPs available for analysis after QC parameters were applied at the level of the individual studies and across all studies contributing to the discovery meta-analysis. Lambda indicates the genomic inflation factor. ACE-I, angiotensin-converting enzyme inhibitor; dCCB, dihydropyridine calcium channel blocker; TD, thiazide diuretic. | | | |

| **SUPPLEMENTAL TABLE 6 Top interaction results of the meta-analysis for left ventricular functions (secondary outcomes) for three antihypertensive medication comparisons** | | | | | | | | | |  |
| --- | --- | --- | --- | --- | --- | --- | --- | --- | --- | --- |
| **RSID** | **Chr:BP** | **A1/A2** | **AF** | **Interaction Effect (SE)** | **Direction** | **P-value** | **Location** | **Gene** | **Model** |  |
| rs11744698 | 5:49917615 | C/G | 0.6967 | 3.5555 | ++ | 7.59E-08 | Intergenic | *PARP8* | GLS dCCB vs. TD |  |
| rs6898102 | 5:49910087 | A/G | 0.6959 | 3.5296 | ++ | 8.18E-08 | Intergenic | *PARP8* | GLS dCCB vs. TD |  |
| rs1562462 | 3:136880567 | T/C | 0.7199 | 3.5577 | ++ | 1.25E-07 | Intergenic | *PPP2R3A* | GLS dCCB vs. TD |  |
| rs1447607 | 3:136880406 | C/G | 0.7208 | 3.5621 | ++ | 1.27E-07 | Intergenic | *PPP2R3A* | GLS dCCB vs. TD |  |
| rs1348975 | 3:136881666 | C/G | 0.1778 | -3.5465 | -- | 1.82E-07 | Intergenic | *PPP2R3A* | GLS dCCB vs. TD |  |
| rs9857474 | 3:136908456 | A/G | 0.1757 | -3.5133 | -- | 2.09E-07 | Intergenic | *PPP2R3A* | GLS dCCB vs. TD |  |
| rs6791230 | 3:136905853 | A/G | 0.1757 | -3.509 | -- | 2.17E-07 | Intergenic | *PPP2R3A* | GLS dCCB vs. TD |  |
| rs1542209 | 3:136901499 | T/C | 0.1768 | -3.4837 | -- | 2.54E-07 | Intergenic | *PPP2R3A* | GLS dCCB vs. TD |  |
| rs9826170 | 3:136903859 | C/G | 0.7176 | 3.4817 | ++ | 2.65E-07 | Intergenic | *PPP2R3A* | GLS dCCB vs. TD |  |
| rs4897664 | 8:135099453 | A/C | 0.2568 | 0.4349 | ++ | 1.77E-07 | Intergenic | *ZFAT/ST3GAL1* | average E/e' ACE-I vs. dCCB |  |
| rs4897665 | 8:135099526 | T/G | 0.6947 | -0.4349 | -- | 1.77E-07 | Intergenic | *ZFAT/ST3GAL1* | average E/e' ACE-I vs. dCCB |  |
| rs16905051 | 8:135098566 | T/C | 0.2635 | 0.4258 | ++ | 3.20E-07 | Intergenic | *ZFAT/ST3GAL1* | average E/e' ACE-I vs. dCCB |  |
| rs4897663 | 8:135097283 | A/G | 0.676 | -0.4237 | -- | 4.02E-07 | Intergenic | *ZFAT/ST3GAL1* | average E/e' ACE-I vs. dCCB |  |
| rs4897662 | 8:135097129 | A/C | 0.6757 | -0.4237 | -- | 4.04E-07 | Intergenic | *ZFAT/ST3GAL1* | average E/e' ACE-I vs. dCCB |  |
| rs4897660 | 8:135096741 | T/C | 0.6756 | -0.4236 | -- | 4.07E-07 | Intergenic | *ZFAT/ST3GAL1* | average E/e' ACE-I vs. dCCB |  |
| rs4897661 | 8:135096847 | A/G | 0.6756 | -0.4236 | -- | 4.08E-07 | Intergenic | *ZFAT/ST3GAL1* | average E/e' ACE-I vs. dCCB |  |
| rs4897626 | 8:135100851 | T/G | 0.2484 | 0.417 | ++ | 1.44E-06 | Intergenic | *ZFAT/ST3GAL1* | average E/e' ACE-I vs. dCCB |  |
| rs10505615 | 8:135102244 | T/C | 0.256 | 0.4053 | ++ | 2.00E-06 | Intergenic | *ZFAT/ST3GAL1* | average E/e' ACE-I vs. dCCB |  |
| rs4897669 | 8:135099919 | T/C | 0.2559 | 0.3973 | ++ | 2.85E-06 | Intergenic | *ZFAT/ST3GAL1* | average E/e' ACE-I vs. dCCB |  |
| rs4897666 | 8:135099721 | A/G | 0.2557 | 0.3973 | ++ | 2.85E-06 | Intergenic | *ZFAT/ST3GAL1* | average E/e' ACE-I vs. dCCB |  |
| rs4897667 | 8:135099762 | A/C | 0.2557 | 0.3973 | ++ | 2.86E-06 | Intergenic | *ZFAT/ST3GAL1* | average E/e' ACE-I vs. dCCB |  |
| rs4897672 | 8:135100645 | A/T | 0.7162 | -0.3972 | -- | 2.87E-06 | Intergenic | *ZFAT/ST3GAL1* | average E/e' ACE-I vs. dCCB |  |
| rs4897671 | 8:135100621 | T/G | 0.7162 | -0.3972 | -- | 2.87E-06 | Intergenic | *ZFAT/ST3GAL1* | average E/e' ACE-I vs. dCCB |  |
| rs11744698 | 5:49917615 | C/G | 0.6967 | 3.5555 | ++ | 7.59E-08 | Intergenic | *PARP8* | GLS dCCB vs. TD |  |
| rs6898102 | 5:49910087 | A/G | 0.6959 | 3.5296 | ++ | 8.18E-08 | Intergenic | *PARP8* | GLS dCCB vs. TD |  |
| RSID, SNP identification; AF, allele frequency; BP, base-pair position; Chr, chromosome; SE, standard error; LVM, left ventricular mass; RWT, relative wall thickness; ACE-I, angiotensin-converting enzyme inhibitor; dCCB, dihydropyridine calcium channel blocker; TD, thiazide diuretic. Direction of effect by individual study were listed in order from left to right as CARDIA and HyperGEN. | | | | | | | | | |  |

**REFERENCES**

Abdalla, M., Booth, J. N., 3rd, Diaz, K. M., Sims, M., Muntner, P., & Shimbo, D. (2016). Hypertension and alterations in left ventricular structure and geometry in African Americans: the Jackson Heart Study. *J Am Soc Hypertens, 10*(7), 550-558 e510. doi:10.1016/j.jash.2016.05.010

Appiah, D., Schreiner, P. J., Gunderson, E. P., Konety, S. H., Jacobs, D. R., Jr., Nwabuo, C. C., . . . Gidding, S. S. (2016). Association of Gestational Diabetes Mellitus With Left Ventricular Structure and Function: The CARDIA Study. *Diabetes Care, 39*(3), 400-407. doi:10.2337/dc15-1759

Arnett, D. K., Devereux, R. B., Rao, D. C., Li, N., Tang, W., Kraemer, R., . . . Broeckel, U. (2009). Novel genetic variants contributing to left ventricular hypertrophy: the HyperGEN study. *J Hypertens, 27*(8), 1585-1593. doi:10.1097/HJH.0b013e32832be612

Arnett, D. K., Meyers, K. J., Devereux, R. B., Tiwari, H. K., Gu, C. C., Vaughan, L. K., . . . Kardia, S. L. (2011). Genetic variation in NCAM1 contributes to left ventricular wall thickness in hypertensive families. *Circ Res, 108*(3), 279-283. doi:10.1161/CIRCRESAHA.110.239210

Daniels, P. R., Kardia, S. L., Hanis, C. L., Brown, C. A., Hutchinson, R., Boerwinkle, E., . . . Genetic Epidemiology Network of Arteriopathy, s. (2004). Familial aggregation of hypertension treatment and control in the Genetic Epidemiology Network of Arteriopathy (GENOA) study. *Am J Med, 116*(10), 676-681. doi:10.1016/j.amjmed.2003.12.032

de Simone, G., Gottdiener, J. S., Chinali, M., & Maurer, M. S. (2008). Left ventricular mass predicts heart failure not related to previous myocardial infarction: the Cardiovascular Health Study. *Eur Heart J, 29*(6), 741-747. doi:10.1093/eurheartj/ehm605

Devereux, R. B., Casale, P. N., Eisenberg, R. R., Miller, D. H., & Kligfield, P. (1984). Electrocardiographic detection of left ventricular hypertrophy using echocardiographic determination of left ventricular mass as the reference standard. Comparison of standard criteria, computer diagnosis and physician interpretation. *J Am Coll Cardiol, 3*(1), 82-87.

Fox, E. R., Klos, K. L., Penman, A. D., Blair, G. J., Blossom, B. D., Arnett, D., . . . Mosley, T. H., Jr. (2010). Heritability and genetic linkage of left ventricular mass, systolic and diastolic function in hypertensive African Americans (From the GENOA Study). *Am J Hypertens, 23*(8), 870-875. doi:10.1038/ajh.2010.67

Fox, E. R., Musani, S. K., Barbalic, M., Lin, H., Yu, B., Ogunyankin, K. O., . . . Vasan, R. S. (2013). Genome-wide association study of cardiac structure and systolic function in African Americans: the Candidate Gene Association Resource (CARe) study. *Circ Cardiovasc Genet, 6*(1), 37-46. doi:10.1161/CIRCGENETICS.111.962365

Fox, E. R., Sarpong, D. F., Cook, J. C., Samdarshi, T. E., Nagarajarao, H. S., Liebson, P. R., . . . Taylor, H. A., Jr. (2011). The relation of diabetes, impaired fasting blood glucose, and insulin resistance to left ventricular structure and function in African Americans: the Jackson Heart Study. *Diabetes Care, 34*(2), 507-509. doi:10.2337/dc10-0838

Fried, L. P., Borhani, N. O., Enright, P., Furberg, C. D., Gardin, J. M., Kronmal, R. A., . . . et al. (1991). The Cardiovascular Health Study: design and rationale. *Ann Epidemiol, 1*(3), 263-276.

Friedman, G. D., Cutter, G. R., Donahue, R. P., Hughes, G. H., Hulley, S. B., Jacobs, D. R., Jr., . . . Savage, P. J. (1988). CARDIA: study design, recruitment, and some characteristics of the examined subjects. *J Clin Epidemiol, 41*(11), 1105-1116.

Fuqua, S. R., Wyatt, S. B., Andrew, M. E., Sarpong, D. F., Henderson, F. R., Cunningham, M. F., & Taylor, H. A., Jr. (2005). Recruiting African-American research participation in the Jackson Heart Study: methods, response rates, and sample description. *Ethn Dis, 15*(4 Suppl 6), S6-18-29.

Gardin, J. M., Siscovick, D., Anton-Culver, H., Lynch, J. C., Smith, V. E., Klopfenstein, H. S., . . . Manolio, T. A. (1995). Sex, age, and disease affect echocardiographic left ventricular mass and systolic function in the free-living elderly. The Cardiovascular Health Study. *Circulation, 91*(6), 1739-1748.

Gardin, J. M., Wagenknecht, L. E., Anton-Culver, H., Flack, J., Gidding, S., Kurosaki, T., . . . Manolio, T. A. (1995). Relationship of cardiovascular risk factors to echocardiographic left ventricular mass in healthy young black and white adult men and women. The CARDIA study. Coronary Artery Risk Development in Young Adults. *Circulation, 92*(3), 380-387.

Harman, J., Walker, E. R., Charbonneau, V., Akylbekova, E. L., Nelson, C., & Wyatt, S. B. (2013). Treatment of hypertension among African Americans: the Jackson Heart Study. *J Clin Hypertens (Greenwich), 15*(6), 367-374. doi:10.1111/jch.12088

Investigators, F. (2002). Multi-center genetic study of hypertension: The Family Blood Pressure Program (FBPP). *Hypertension, 39*(1), 3-9.

Keku, E., Rosamond, W., Taylor, H. A., Jr., Garrison, R., Wyatt, S. B., Richard, M., . . . Sarpong, D. (2005). Cardiovascular disease event classification in the Jackson Heart Study: methods and procedures. *Ethn Dis, 15*(4 Suppl 6), S6-62-70.

Kishi, S., Reis, J. P., Venkatesh, B. A., Gidding, S. S., Armstrong, A. C., Jacobs, D. R., Jr., . . . Lima, J. A. (2015). Race-ethnic and sex differences in left ventricular structure and function: the Coronary Artery Risk Development in Young Adults (CARDIA) Study. *J Am Heart Assoc, 4*(3), e001264. doi:10.1161/JAHA.114.001264

Meyers, K. J., Chu, J., Mosley, T. H., & Kardia, S. L. (2010). SNP-SNP interactions dominate the genetic architecture of candidate genes associated with left ventricular mass in African-Americans of the GENOA study. *BMC Med Genet, 11*, 160. doi:10.1186/1471-2350-11-160

Spring, B., Moller, A. C., Colangelo, L. A., Siddique, J., Roehrig, M., Daviglus, M. L., . . . Liu, K. (2014). Healthy lifestyle change and subclinical atherosclerosis in young adults: Coronary Artery Risk Development in Young Adults (CARDIA) study. *Circulation, 130*(1), 10-17. doi:10.1161/CIRCULATIONAHA.113.005445

Tang, W., Devereux, R. B., Rao, D. C., Oberman, A., Hopkins, P. N., Kitzman, D. W., & Arnett, D. K. (2002). Associations between angiotensinogen gene variants and left ventricular mass and function in the HyperGEN study. *Am Heart J, 143*(5), 854-860.

Williams, R. R., Rao, D. C., Ellison, R. C., Arnett, D. K., Heiss, G., Oberman, A., . . . Hunt, S. C. (2000). NHLBI family blood pressure program: methodology and recruitment in the HyperGEN network. Hypertension genetic epidemiology network. *Ann Epidemiol, 10*(6), 389-400.
